# Modeling *Drosophila* Positional Preferences in Open Field Arenas with Directional Persistence and Wall Attraction

**DOI:** 10.1371/journal.pone.0046570

**Published:** 2012-10-10

**Authors:** Benjamin Soibam, Rachel L. Goldfeder, Claire Manson-Bishop, Rachel Gamblin, Scott D. Pletcher, Shishir Shah, Gemunu H. Gunaratne, Gregg W. Roman

**Affiliations:** 1 Department of Computer Science, University of Houston, Houston, Texas, United States of America; 2 Department of Biology and Biochemistry, University of Houston, Houston, Texas, United States of America; 3 University of Michigan Geriatrics Center, Department of Molecular and Integrative Physiology, University of Michigan, Ann Arbor, Michigan, United States of America; 4 Department of Physics, University of Houston, Houston, Texas, United States of America; 5 Biology of Behavior Institute, University of Houston, Texas, United States of America; Alexander Flemming Biomedical Sciences Research Center, Greece

## Abstract

In open field arenas, *Drosophila* adults exhibit a preference for arena boundaries over internal walls and open regions. Herein, we investigate the nature of this preference using phenomenological modeling of locomotion to determine whether local arena features and constraints on movement alone are sufficient to drive positional preferences within open field arenas of different shapes and with different internal features. Our model has two components: directional persistence and local wall force. In regions far away from walls, the trajectory is entirely characterized by a directional persistence probability, 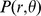, for each movement defined by the step size, 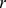, and the turn angle, 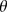. In close proximity to walls, motion is computed from 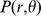 and a local attractive force which depends on the distance between the fly and points on the walls. The directional persistence probability was obtained experimentally from trajectories of wild type *Drosophila* in a circular open field arena and the wall force was computed to minimize the difference between the radial distributions from the model and *Drosophila* in the same circular arena. The two-component model for fly movement was challenged by comparing the positional preferences from the two-component model to wild type *Drosophila* in a variety of open field arenas. In most arenas there was a strong concordance between the two-component model and *Drosophila*. In more complex arenas, the model exhibits similar trends, but some significant differences were found. These differences suggest that there are emergent features within these complex arenas that have significance for the fly, such as potential shelter. Hence, the two-component model is an important step in defining how *Drosophila* interact with their environment.

## Introduction

Locomotion is a central component of many behaviors including searching for food, shelter, and escape routes. Careful analysis of locomotion may also be used to infer some features of complex behavioral characteristics such as memory [1]–[4], sleep [5]–[7], anxiety [8]–[12], reaction to novelty [13]–[16], courtship [17]–[19], and drug addiction [20]–[22]. However, animal locomotion is a multi-factorial phenomenon. Each trajectory is unique and involves interactions between the animal's nervous system and the environment [23], [24]. Consequently, decomposing the motion to its constituents, and deducing the rules underlying motion become non-trivial tasks. The goal of our work is to outline an approach that combines experimental data and phenomenological modeling, to attain these goals.

Controlled laboratory studies on animal locomotion are frequently conducted in open field arenas with regular geometries [25]. Statistical features of the motion, such as path length distributions or the speed and turn angles are quantified using a large ensemble of trajectories [23]. Temporal variations in these characteristics can be used to infer behavioral changes in animals, for example, as the novelty of an arena is abrogated [16].

*Drosophila melanogaster*, similar to most animals, display a significant preference for the arena's edge in open field arenas [16], [23], [26]–[28]. Two proposals for this observation include an innate desire to be near walls. Centrophobicity asserts that animals avoid central regions of an arena and spend time near walls, perhaps due to an absence of cover against predators [26], [27]. Centrophobicity may not be the primary driving force for edge affinity because blind flies also display significant wall-following behavior [29]. Thigmotaxis proposes an animal's affinity for the boundary is due to arousal gained by contact with walls [26], [27]. However *Drosophila* do not prefer all walls equally, favoring boundaries over internal walls [29], arguing against a simple thigmotactic motivation. It has been further suggested that prevalence of straight trajectories with a failure to make frequent sharp turns would promote a high occupancy at the boundaries of arenas [30], but persistent straight trajectories was by itself is insufficient to account for the movement and positional preferences of *Drosophila* in an hourglass-shaped arena [29]. Specifically, flies display wall-following behavior even along convex curves in an hourglass-shaped arena, indicating that walls offer attractive features which contribute significantly to positional preference of a fly inside open field arenas [29].

This attraction to arena walls can be a response to both the local and global features of the arena [31]–[35]. Previous studies have proposed that flies actively explore the arena boundary through proximate investigation [31], [33], [35], even though flies can also detect and respond to distal cues [31], [32], [34]. Herein, we further investigate the role of local wall attraction and directional persistence for driving edge preference through phenomenological modeling of this behavior. In our model, locomotion of the fly is controlled by two rules: (1) in regions far away from walls, its motion is stochastic and determined from a directional persistence probability, 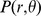, of each movement defined by the step size, *r* and the turn angle, 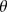, as measured from wild type flies within a circular arena of radius 4.2 cm;(2) it is attracted to the walls with a force which depends on the distance between the fly and nearby walls. Items (1) and (2), and the constraints imposed by the arena boundary completely define locomotion inside open field arenas. The characteristics of the two-component model indicate that the fly is not centrophobic, does not search for escape hatches in the arena, has no intrinsic preference for corners, and does not differentiate between internal and external walls. In this model, animal locomotion within the arena is primarily determined by actions specified locally and with no significant consideration to global facets of an arena. The accuracy of this two-component model is tested by comparing the statistical properties of *Drosophila melanogaster* trajectories with those obtained from the two-component model. Consistent with the behavior of the wild type fly, the two-component model exhibits, what appears to be, an affinity for corners [23] and a preference for external walls over internal walls [29], even though these facets were not explicitly built in to its construction. Inside an hourglass-shaped arena, the trajectories from the two-component model display similar wall-following behavior to that of *Drosophila*. Since the two-component model accurately predicts positional preferences in several different arenas, it suggests that directional persistence and local wall force attraction contribute to positional preferences in *Drosophila*. Moreover, our results indicate that wild type flies primarily attend to local stimuli. In arenas with more complex geometry, the model captures several trends for specific spatial preferences in different arenas but underemphasizes the overall preference for boundary zones exhibited by wild type *Drosophila*. These results may be consistent with *Drosophila* relying on both proximal and distant cues during exploration of an open field arena. Finally, comparing simulations and experiments with mutant flies with wall-following defects and, hence, altered movement parameters, we show that intrinsic processes exist which affect the nature of the fly's response to local and distant cues in an open field arena.

## Materials and Methods

### Fly stocks and husbandry

All stocks were raised and maintained on standard yeast-cornmeal agar food at room temperature. Flies that were used in behavioral assays were raised on standard food at 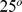
C, 60% humidity, with 12 hr of light/dark. The 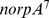 mutants were obtained from the Bloomington Stock Center. The 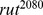 and 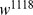 mutations were obtained from Ronald Davis (Scripps FL). The 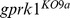 mutants were obtained from David Hipfner (McGill University). The 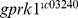 mutants were a generous gift from Paul Hardin (Texas A & M University). The mutations were all crossed into a wild type Canton-S genotype for a minimum of 6 generations.

### Behavior Assays and Discretization of Trajectories

The first set of arenas used in our study were made of transparent plexiglass by the University of Houston Physics Machine shop. The experimental data from circular, square, concentric circle, internal corner and hourglass-shaped arena were previously used in Ref. [29]. As previously described in Ref. [29], the height of all these five arenas was 0.7 cm. The length of each side of the square arena was 7.2 cm. The radius of the circular arena, the concentric circle arena, and internal corner arena was 4.2 cm. In the internal corner arena, the perpendicular intersecting walls extended 3.2 cm from the center. There was a 1 cm gap between the wall and the circular boundary. In the concentric circle arena with internal concentric walls (4.2 cm radius), the concentric walls were equally spaced. The hourglass-shaped arena was 10 cm long and 6 cm wide with a 2 cm wide central gap.

The spiral and the Texas arenas were designed in two dimensions in Adobe illustrator (San Jose, CA) and printed in three dimensions with a depth of 0.7 cm using a Dimension 1200 es Printer (Dimension, Inc. Eden, MN). The spiral arenas were 9 cm in length across the longest axis. The Texas-shaped arena was 9 cm in the North-South axis. These arenas were composed of a white, opaque thermoplastic. A circular arena of 4.2 cm radius and 0.7 cm height was also printed and used to calibrate the movement parameters of wild type Canton-S flies in the printed arenas with opaque white walls.

**Figure 1:**
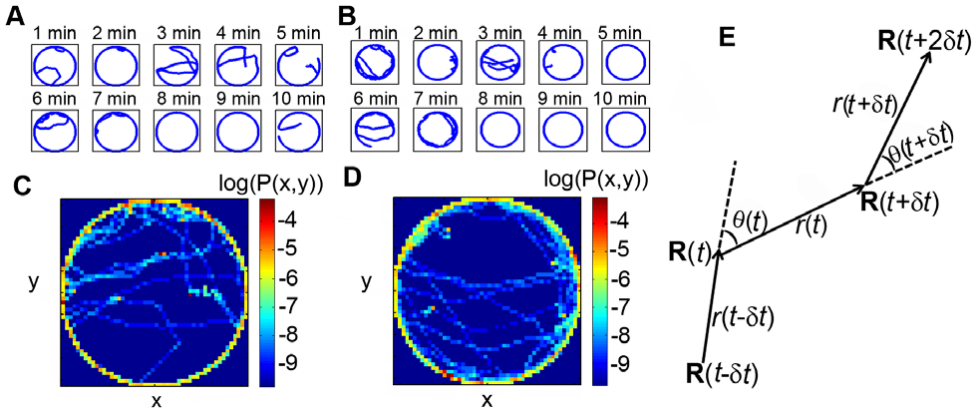
Position characteristics of a fly inside a circular arena of radius 4.2 cm. Two experiments, each of 10 minute duration broken into 1 minute intervals, are shown in **A** and **B**. The spatial density, P(x,y), of the fly in **A** and **B** are shown in **C** and **D**, respectively, in logarithmic scale. The density plots clearly indicate that the fly prefers the boundary and has no angular preference along the boundary. In panel **E**, we illustrate turn angle, 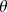, and step size, 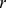, in the two-component model. There are four consecutive locations of the fly at time 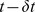, 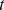, 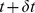 and 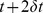. Turn angle 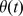 at time 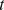 is measured with respect to the direction of movement at the previous time step. The direction of movement at time 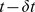 is along the direction of vector 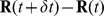. The step length 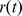 at time 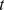 is given by 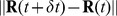.

As previously described by Ref. [29], the tops of the arenas were lids of 15-cm petri plates (Fisher Scientific). A 2-mm hole was drilled in the top to allow for the aspiration of a fly into the arena. Since the top was larger than the arena, the hole could be shifted out of the active arena area once the fly was introduced. The arena was illuminated by two 23 W compact fluorescent flood lights (R40, 1200 lumens, 5100 K). Ethovision XT v5.0 (Noldus Information Technology, Leesburg VA) was used to track the movement of the fly within the arena at a recording rate of 30 frames per second. The resulting trajectory was smoothed using a running line regression with a window of 5 data points (0.2 s) and a 1 point step size (0.04 s) [28]. Position characteristics of a fly inside a circular arena of radius 4.2 cm are shown in Figures 1A–D. Trajectory plots of Canton-S flies in other arenas are shown in Figures S1, S2, S3, S4.

In this paper, we disregard the time dependencies in motion, and only consider time-averaged statistical characteristics. Each trajectory was discretized with a time-unit 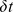 of 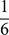 second, and motion within this time interval was assumed to be linear. The time unit is chosen so that, typically, the animal's motion during the interval is close to a straight line and so that the typical displacements are significantly larger than measurement errors.

### Directional Persistence

Positions at three consecutive time-points were used to calculate the turn angle. Given the three vector locations 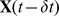, 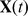 and 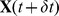 of a fly trajectory at times 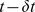, 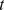 and 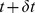, the turn angle, 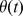, at time 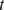 (See Figure 1E) is computed from the cosine rule
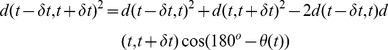
(1)Here 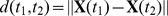 is the distance traveled during the time interval from 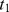 to 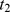. The time interval 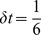 second. The step size at time 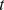 is 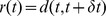 and 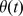 is the change of orientation during successive time steps 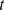 and 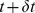. Directional persistence is quantified through the probability 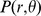, which is computed using displacements 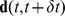 over all the trajectories and referred to as the directional persistence probability. However, since the wall effects need to be avoided, we only included those points for which both 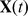 and 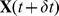 are in the central zone of the circular arena with a 4.2 cm radius. The central zone is a circular region inside the circular arena where the wall effects are assumed to be non-existent. In addition, we assume symmetry about 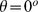, and hence only compute 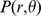 for 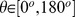.

### The Model

The phenomenological model introduced here is used to assess whether components of *Drosophila* movement alone can account for the signficant positional preferences within arenas. The model is tested by comparing statistical features of the trajectories from the model and *Drosophila* in a wide array of semi-regular and irregular arenas. These open field arenas, imported into our simulations, were drawn manually using Adobe Illustrator and saved as a JPEG image. The arena was generated as a two dimensional lattice of nodes. The nodes can be either wall nodes or potential positions of the fly inside the arena. The set of wall nodes comprised of all the dark pixels in the JPEG image which had intensities lower than a certain threshold. The fly's motion is restricted to the interior of this boundary.

A trajectory in regions sufficiently away from walls is modeled by a set of stochastic events representing its motion in time units of 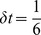 second. We assume that the environmental conditions are uniform for the duration of a trajectory, and model each step via the step size, 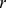, and the turning angle, 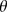 [36]–[40]. Each step is chosen randomly from the directional persistence probability 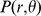, where, as discussed earlier, 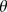 is measured relative to the direction of the previous step.

A large fraction of *Drosophila* trajectories in circular arenas are executed near the external walls [16], [26]–[28]. We model this wall affinity by an attractive force from sites sufficiently close to the wall. In order to define the force, we introduce a comfort zone for the fly in the two-component model. Specifiï¿½cally, it is a semi-circular area of radius, 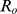, centered at the current location of the fly and centered along the direction of its motion. We assume that only the wall nodes within the comfort zone of the fly affect its motion. The attractive force from each such node on the fly is assumed to decrease linearly as the distance from the node increases; *i.e.,*

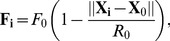
(2)where 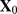 and 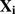 are the locations of the fly and the wall node, and 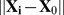, assumed to be less than 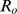, is its distance between the points. The force vanishes when 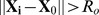. The force on the fly is directed towards the wall node 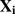. Observe that this interaction reduces smoothly to zero at the edge of the animal's comfort zone. We find that the precise form of the monotonically decreasing function does not affect the statistical features of the animal's trajectories, as long as the value of 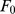 is appropriately chosen. The total force on the fly from the wall is
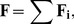
(3)the summation being over all the wall nodes in the fly's comfort zone.

There are three assumptions for the wall attraction in the two-component model. They are: (1) the wall attractive force has a magnitude that decreases linearly with the distance between the fly and wall node, (2) the wall attraction is damping, and (2) the wall attractive force vanishes when the distance between the fly and a wall node is greater than radius of the comfort zone. To explore other forms of force, we also considered two forms of a nonlinear attractive force (equations 5 and 6) between the wall node and fly.

#### Trajectories from the two-component model

The motion in the two-component model is entirely governed by a (1) stochastic process given by 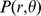 in the central zone, and (2) an attraction given by Equation (2) to each wall node in the animal's comfort zone. Each trajectory is initiated inside an arena and the fly's initial direction is chosen randomly. The comfort zone of the fly in the two-component model is computed from its location and orientation. The movement of the fly in the two-component model was the summation of 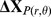, a random variable from 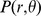, and 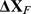, the wall effect. We assume that the animal's dynamics is damping dominated and hence that 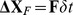, where 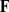 is given in Equation (3). If the resultant location of the fly falls in an unreachable region (*e.g.*: outside the arena, or unreachable node because of internal walls, etc.), a repulsive force 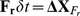 keeps the fly inside the arena or prevents crossing internal walls. The force 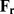 is along the direction to the nearest reachable node from the current unreachable node and the magnitude is the distance between the current unreachable node and the nearest reachable node.

### Model validation

**Figure 2:**
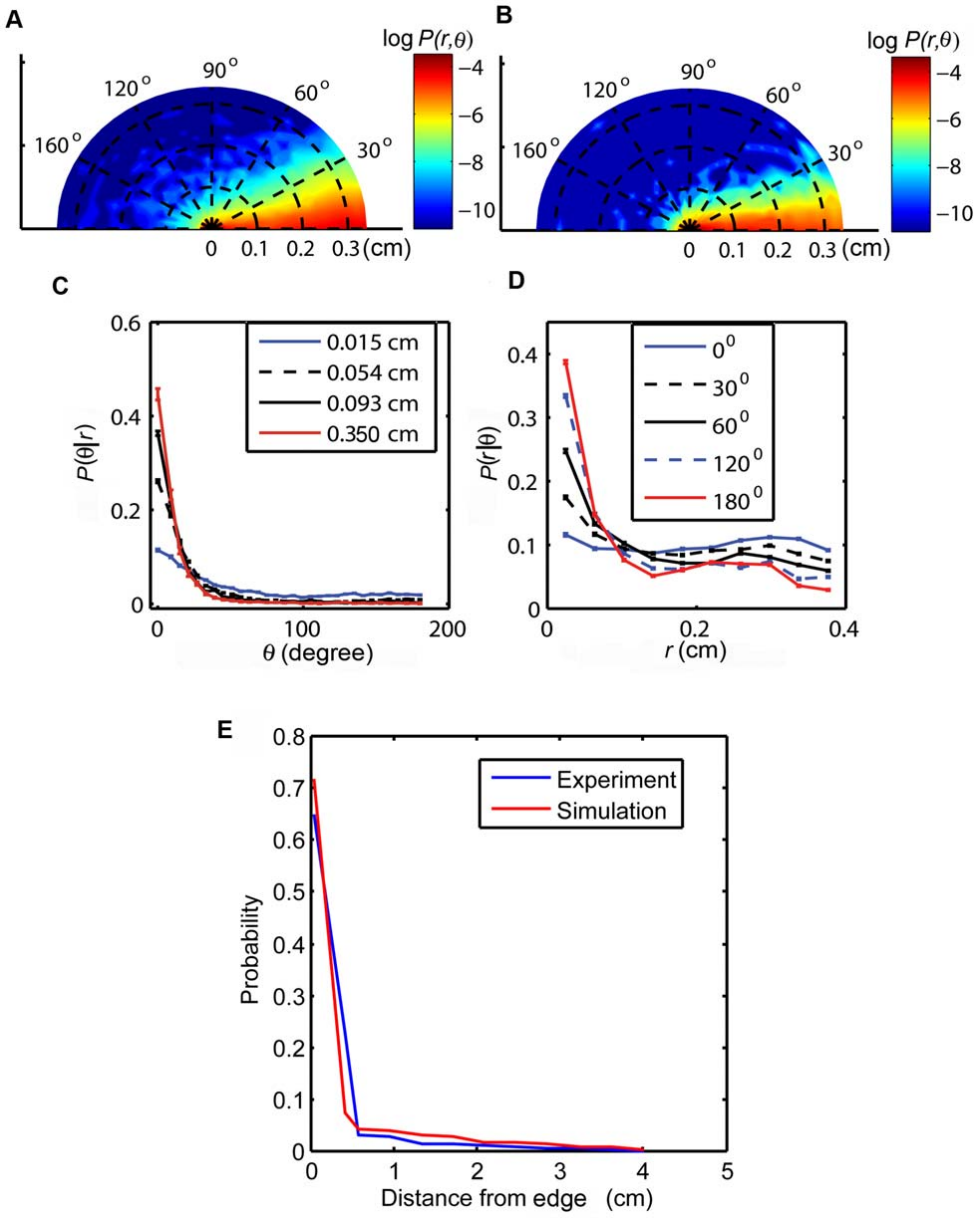
Mean occupancy in different sections of the 7.2 cm×7.2 cm square arena. **A.** The 7.2 cm square arena was divided into 16 equal 3.24 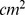 sectors. These sectors were characterized to three different zones: corner (4 sectors), wall (8 sectors), and center (4 sectors). **B.** The mean occupancies of the flies, both from simulations and experiments, in each zone are shown. The times spent in these zones by *Drosophila* were statistically similar to that from the two-component model.

**Figure 3:**
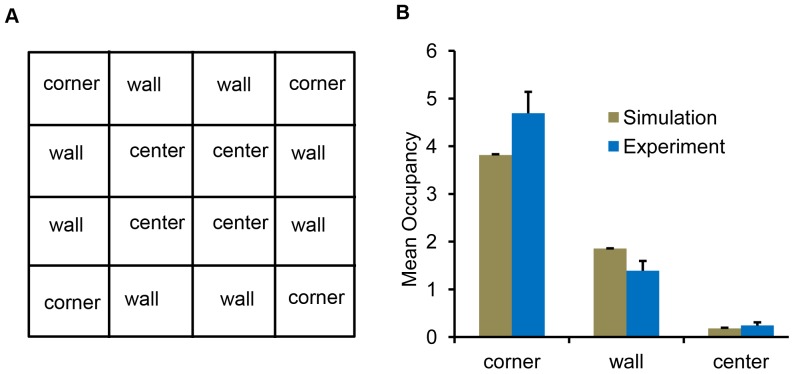
Mean occupancy in different zones of 4.2 cm circular arena with internal corners. **A.** The internal corner arena was constructed with a cross placed in the center of a 4.2 cm circular arena. We considered two zones in this arena: cross and edge zone. The cross zone was a 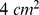 square sector positioned at the center while the edge zone was a annular region of width 0.6 cm along the boundary. **B.** The mean occupancies of the flies, both from simulations and experiments, in each zone are shown. The times spent in these zones by *Drosophila* were statistically similar to that from the two-component model. *Drosophila* showed a preference for the boundary over internal corners.

**Figure 4:**
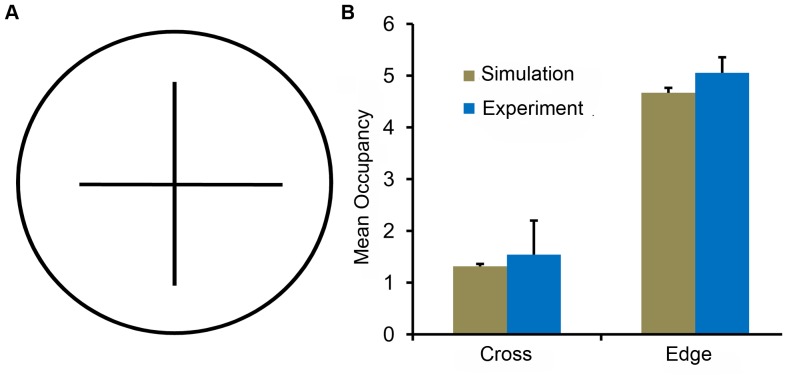
Mean occupancy in different zones of a concentric circular arena. **A.** An arena was constructed with internal concentric walls. For analysis, the arena was subdivided into 4 zones: zone 1, zone 2, zone 3, and zone 4. **B**. The behavior of flies was examined in the concentric circle arena. The mean occupancies in four different zones, both from simulations and experiments, are shown. *Drosophila* showed a preference for the outermost zone. The times spent in these zones by *Drosophila* were statistically similar to that from the two-component model.

**Figure 5:**
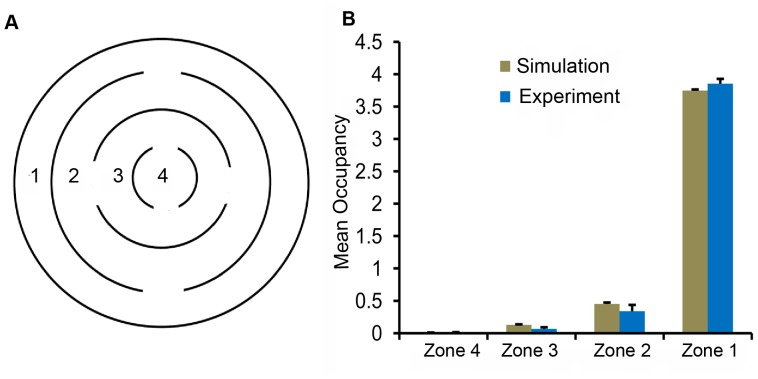
Vertical and horizontal transitions inside an hourglass-shaped arena. **A.** The hourglass-shaped arena is shown. A fly walking in this arena may make a horizontal transition (H.T) by following the wall from one chamber to the next, or it may make a vertical transition (V.T) by crossing the central gap. **B**. The vertical transition indices, both from simulations and experiments, are shown. There was no significant difference between the experiments and simulations. *Drosophila* showed more horizontal transitions.

In our study, we used the trajectories of a fly in a circular arena of 4.2 cm radius to estimate the model parameters; 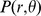, 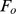 and 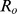. The estimated parameters were used to simulate the trajectories inside four other arenas: square arena (Figure 2), internal corner arena (Figure 3), concentric circle arena (Figure 4), and hourglass-shaped arena (Figure 5). To validate the two-component model, we divided each of the four arenas into distinct spatial zones. The occupancies (the ratio of % of time spent in a zone to the area of the zone) in a zone computed from these experiments and the simulations were compared statistically. We then tested the model using four different mutant genotypes, which had shown altered wall affinity [16], [41]. The model parameters for each of the mutants were estimated using their trajectories in the circular arena of radius 4.2 cm. The simulated trajectories of the mutant flies inside two arenas (square arena (Figure 2)and internal corner arena (Figure 3)) were compared with those from experiments. Finally, the two-component model predictions of spatial preference of wild type flies inside more complex arenas, an irregular Texas-shaped arena and two spiral arenas, were checked against those from experiments. The model parameters for these arenas were estimated using the trajectories of Canton-S flies in the opaque thermoplastic circular arena of radius 4.2 cm. The threshold of 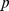 value used in this paper was 0.05. We used ‘occupancy’ instead of ‘percentage of time’ spent in zones to test the two-component model because the zones in many arenas had different areas and using ‘percentage of time spent’ in zones can give biased preferences for zones with higher areas. Occupancy allows comparison of positional preferences of different zones having different areas.

## Results

**Figure 6:**
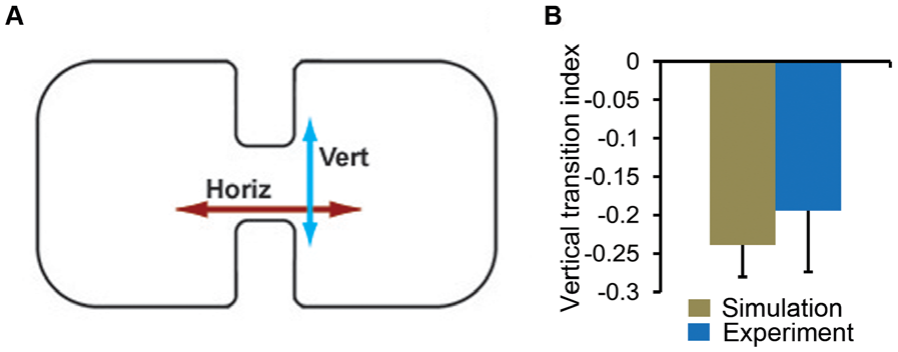
Analysis of directional persistence probability and wall attraction. **A**. 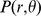 in the central zone of a circular arena of radius 4.2 cm is shown. 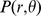 was computed with a bin size of 0.18 cm along 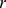 and 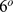 along 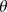. A cell in the two dimensional plot represents a pair of 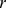 and 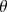. **B**. 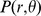 in the central zone of a circular arena of radius 2.5 cm is shown. The trajectories of the fly in the central zone of the two circular arenas were similar. **C**. 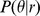 in the central zone of radius 4.2 cm arena. To obtain 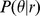 for 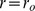, portions of the trajectories with radial displacements, 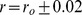, cm were considered. **D**. 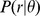 in the central zone of radius 4.2 cm arena. To obtain 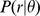 for 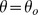, portions of the trajectories with angular displacements, 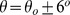, were considered. **E**. Radial distribution of a wild type fly and simulations, with 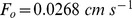, in the circular arena of radius 4.2 cm. The radial distribution from the simulations with 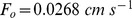 was closest to that of *Drosophila*.

In circular arenas, the interface between central and edge zones can only depend on the radial distance 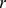, or equivalently the distance 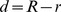 from the wall, where 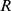 is the radius of the arena. Previous studies have shown that *Drosophila* adults prefer locations which are close to the edge [23], [26], [28]. In order to quantify this behavior, and to infer the interface between the zones, we use the (time-averaged) radial distribution function. We also verified that flies had no biased preference for any of the four quandrants in the circular arena (Figure S5). As shown in Figures 1 & 6, flies spent over 90% of the duration of the trajectory within 6 mm from the edge. We thus interpret the 6 mm wide annular regions near the wall to be the edge zone, where the animal's wall affinity is noticeable; the remaining region was assigned to be the central zone.

### Directional persistence

We assume that the spatial isotropy of animal locomotion is only broken by its motion; in this case, 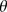, the turn angle, is measured relative to the direction of the immediately preceeding step [38], [40], [42]. The resulting directional persistence is quantified by 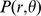, which was estimated using 272 trajectories in the central zone of a circular arena of radius 4.2 cm. It should be emphasized that the form of 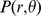 is established experimentally, and that we do not provide a theory for it. The estimated 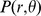 is heavily weighted in the forward direction (Figure 6A). 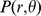 is independent of the radius of the circular arena (Figure 6A & B, 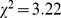, 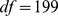, 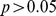). Our analysis also shows that 60%, 72% and 80% of a fly's movement were restricted to turn angles smaller than 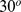, 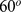, and 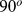, respectively. This indicates the presence of strong forward persistent locomotion within the central zone.

Many studies assume, in addition, that the radial and angular displacements are independent and that 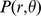 can be decomposed as 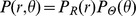, where 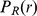 and 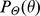 are the radial and angular probability density functions, respectively [36]–[40]. However, the existence of correlations between step length and turn angle has also been noted [43]. If the probability density is decomposable, the (normalized) conditional probability 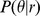 (*i.e.,* projection of 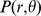 into 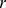) will be independent of 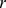. Similarly, normalized projection 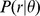 will be independent of 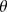. To examine decomposability, we computed 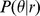 for four different step lengths (Figure 6C). There was a significant effect of 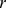 on these distributions (verified by a 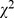 test, shown in Table S1), indicating that the fly's turning characteristics depend on its step size. At any value of 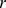, 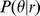, was found to decrease with an increase in turn angle, indicating the animal's preference for maintaining their direction at any speed. We also find that 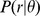 for five different turn angles (Figure 6D) were statistically different (as verified by a 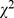 test, shown in Table S2). When the flies maintained their direction (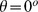), all step lengths within an interval were equally likely. However, when turning by more than 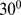, the flies appear to prefer smaller step lengths. This analysis indicates that, at least for *Drosophila*, the assumption of independence between speed and turn angle is invalid. We also verified that the independence between speed and turn angle at different values of 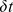 (Figure S6).

### Wall attraction

The intensity of attraction towards the wall nodes, 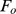, determines the fraction of time a fly spends in the edge zone; larger values of 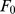 enhance the occupational probability in the edge zone. Hence, we can use the radial distribution function (Figure 6E) to estimate 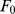. For different values of 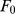, we computed the radial distributions obtained from the two-component model and compared the results with the radial distribution for *Drosophila* (Figure S7). We find that the two distributions are closest when 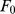 is chosen to be 0.0268 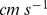 (see Figure 6E and Figure S7).

### Trajectories in Other Arenas

In order to examine the accuracy of the two-component model for *Drosophila* behavior in open field arenas, we compared the positional preferences of wild type and those obtained from the two-component model in a variety of arenas. The parameters estimated from trajectories of *Drosophila* in the circular arena of radius 4.2 cm were used to simulate movements in other arenas. We studied the simulated trajectories in several types of arenas and conducted a statistical comparison with trajectories of *Drosophila*. The arenas were chosen to test specific aspects of locomotion [29].

#### Square arena

The goal of these studies is to establish if the *Drosophila* preference for corners [16], [23] can result from directional persistence and wall attraction alone. In order to quantify this corner preference, we divided the square arena (7.2 cm×7.2 cm) into three different spatial zones: corners, walls and center (Figure 2). The occupancies of the fly in these three zones were compared to corresponding quantities obtained using the two-component model. They were found to be statistically identical in each zone (Figure 2; corner: 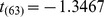, 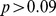; wall: 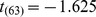, 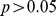; center: 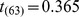, 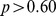). These results are consistent with the corner preference resulting solely from directional persistence and wall attraction. Trajectories in the two-component model experience a higher number of wall nodes near the corners compared to the walls. Therefore, the wall attraction offered by the nodes in the corners can cause the fly to visit the corners more often compared to the walls. Directional persistence will also keep the fly in the corner for longer periods while it changes its trajectory. Our results indicate that such trajectories can generate similar positional preferences to *Drosophila* in the square arena.

#### Internal corner arena

Our next experiments were conducted in a circular arena with internal walls that generate four corners in the center of the arena; see Figure 3. The occupancy of wild type flies near the internal walls was significantly lower than that near the external walls. Specifically, the occupancy in a 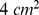 square sector in the the center of the arena was higher (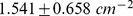), compared to the same zone in an open circular arena (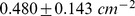), but was lower than in the edge zone (internal corner arena: 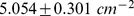 and open circular arena: 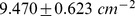). The simulated trajectories, which had no explicitly coded preference for internal vs. external walls, exhibited a similar spatial preference (center: 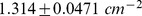 and edge: 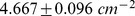). The differences in occupancies for the two-component model and *Drosophila* are statistically insignificant (center: 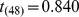, 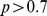, edge: 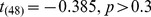). Thus, a local wall force and directional persistence movement are sufficient to explain positional preference in the internal corner arena. It seems likely that the difference in corner extraction found between the internal and external corners is due to the influence of the directional persistence driving outward trajectories.

#### Concentric circular arena

In these experiments, we examined if the concave curvature of the circular exterior wall is specifically attractive for *Drosophila* by providing an arena with several internal curved vertical surfaces. The circular arena of radius 4.2 cm was partitioned into four concentric annular zones (Figure 4A). Moving centrally, each wall has an increased curvature, allowing for a greater density of points along the inner concave walls. Wild type Canton-S flies prefer the outermost zone, suggesting a strong bias of arena boundary and not simply curved walls [29]. Once again, there were insignificant differences between the occupancies of *Drosophila* and those obtained from the two-component model in the four annular zones (Figure 4, zone 1: 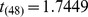, 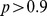; zone 2: 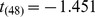, 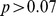; zone 3: 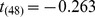, 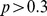; zone 4: 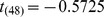, 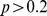). The preference of the arena boundary over internal vertical surfaces can be explained with directional persistence and an unbiased wall attraction. Trajectories which are driven by a local wall attraction and directional persistence can leave zones 1, 2 and 3 through the gaps (Figure 4). Once the flies reach the boundary, the local wall attraction might cause the flies to spend more time along the boundary interrupted by infrequent large radial or angular displacements that can point the trajectories away from the boundary. Our results indicate that such trajectories can give rise to similar positional preferences exhibited by *Drosophila*.

#### Hourglass-shaped arena

An arena shaped like an hourglass can be used to distinguish the strength of active wall-following behavior and directional persistence behavior. The arena, shown in Figure 5, has two chambers joined by a relatively narrow channel. Its edge contains convex and concave sections and can be used to distinguish between active and passive wall-following behavior of *Drosophila*. The narrow channel in the arena creates two possible options for locomotion; animals can continue a linear trajectory (vertical transitions) or execute a wall following motion (horizontal transitions). We counted trajectories that passed the horizontal midpoint of the central gap as horizontal transitions (HT). Those trajectories which crossed the vertical midpoint of the 2 cm central chasm were taken as vertical transitions (VT). A diagonal movement though the chasm was reported as both a horizontal transition and a vertical transition. The choice between these possibilities was quantified using the vertical transition index (VTI),
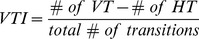
(4)


Movements driven primarily by directional persistence will yield a positive VTI, while those driven by wall attraction will have a negative VTI. The VTI obtained from the two-component model (−0.24±0.04) and *Drosophila* (−0.19±0.08) were statistically similar (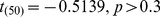); see Figure 5. It has been shown that a model driven by directional persistence alone cannot account for the observed wall-following behavior [29]. Therefore, directional persistence and wall attraction together can produce the observed behaviors in the hourglass arena. Trajectories which are driven by local wall attraction are more likely to follow the walls close to the central chasm resulting in more horizontal transitions than vertical transitions.

The agreement between the statistical properties of the trajectories of wild type *Drosophila* and those from the two-component model in a range of arenas strongly supports our conjecture that (1) movement away from the walls is stochastic and governed by 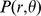, and (2) the flies inside the edge zone experience an attractive force towards the wall. We hypothesize that these are two of the principles that appear to largely govern positional preferences of *Drosophila* in open field arenas.

### Spiral arenas

The success of the two-component model suggests that directional persistence and a local wall force are sufficient to account for the wall-following behavior and preference for the arena's boundary over internal walls. The wall attraction may keep them close to the wall, while the directional persistence may propel them radially toward the boundary. An alternative explanation could be that wild type *Drosophila* use global features of the arena to identify the arena's edge and specifically attend to this feature as the outermost boundary of the territory. To begin discriminating between these two possibilities, we examined the behavior of *Drosophila*, both experimentally and with the two component model, in different spiral arenas. In spiral arenas with continuous walls, the directional persistence would lead the fly to the center rather than stuck at the outermost edge. Since the spiral arenas were of a white, opaque thermoplastic, we examined the trajectories of *Drosophila* in a 4.2 cm circular arena composed of white, opaque thermoplastic. We found that 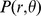 and the radial distribution in this circular arena were not statistically different from the clear 4.2 cm circular arena (Figure S8). Therefore, the same value of 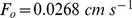 estimated from the clear circular arena was used to simulate movement trajectories in the spiral arenas. We used the directional persistence probabilty, 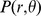, estimated from the white, opaque thermoplastic circular arena (Figure S10).

**Figure 7:**
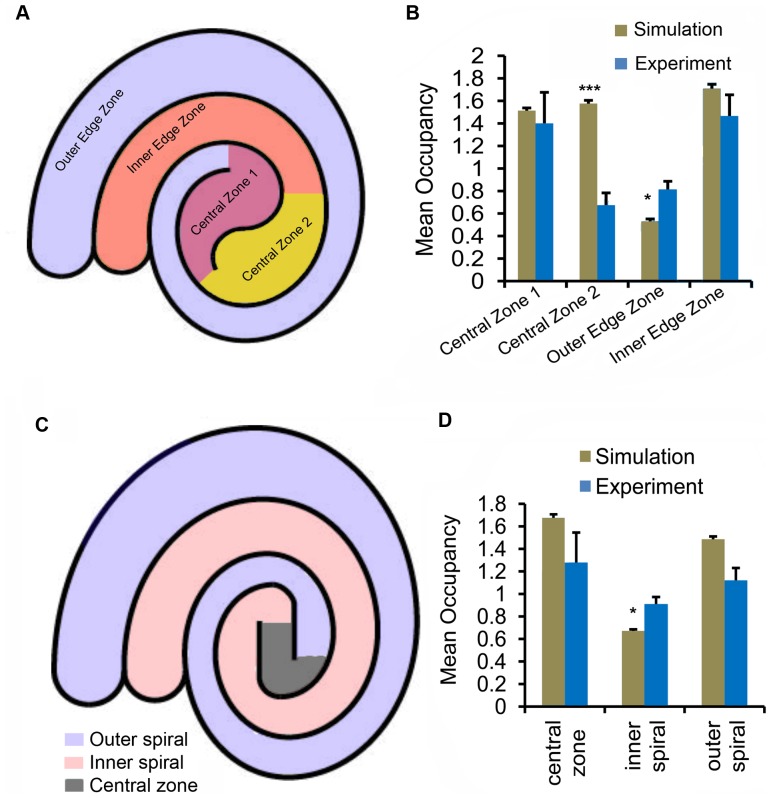
Positional preferences from experiments and simulations in two double spiral arenas. The double spiral arenas are shown in panels **A** and **C**. Each arena was divided into different spatial zones. The mean occupancies in these zones for arena **A** and **C** are shown in **B** and **D**, respectively. In the spiral arenas, the occupancy was computed as a ratio of fraction of total time spent in a zone to the fraction of the total arena occupied by the zone. The asterix indicates significant difference between simulations and experiments (*: 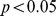, **: 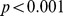, and ***: 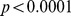).

We designed two double spiral arenas (Figure 7) with differences in the central zones that were predicted to alter occupancy of this zone for flies displaying directional persistence. The continuous wall and increased vertical surfaces found internally in these spiral arenas would be predicted to increase the time spent in the arena center according to the directional persistence and local wall force model; however, if the flies are using a global mapping strategy, they will continue to avoid the center and still show a signficant preference for the outer edge of the spiral arenas. To test our hypothesis, we partitioned the arenas into different spatial zones as shown in Figure 7 and compared the positional preferences of wild type Canton-S and those from the simulations.

In the first double spiral arena, the outer edge zone was less preferred in the simulations as compared to the other three zones (Figure 7). This decreased occupancy of the outer zone may be acccounted for by the concave curvatures of walls surrounding central zone 1, which are expected to bias residence in this zone. Interestingly, our simulations indicate that the mean occupancies in the first central zone and inner edge zone were similar to that of wild type Canton-S flies (Figure 7B; central zone 1: 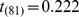, 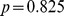; inner edge zone: 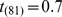, 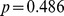). However, our simulations over-estimated the spatial preference of central zone 2 and under-predicted the preference of outer edge zone (Figure 7B; central zone 2: 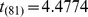, 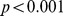; outer edge zone: 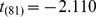, 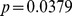). In our experiments in the second double spiral arena, flies preferred the outer spiral zone and the central zone equally (Figure 7C). There were no significant differences in the occupancies of these two zones between experiments and simulations (central zone: 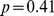, 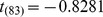; outer spiral: 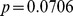, 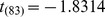). Our simulations underestimated the inner spiral preference (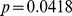, 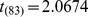). A possible source for the significant differences in positional preferences in these two spiral arenas may be the fact that we have not modeled locomotion at the end of a wall segment. For example, as a fly moves from central zone 2 to central zone 1 in the first double spiral arena, the end of the partitioning wall may act to refract the fly's movement to direct a significantly different turn angle. Another possible explanation is that *Drosophila* use a global mapping strategy to avoid central zones.

We draw two conclusions from our analysis with spiral arenas: (a) the increased preferences of the central zones support the local rules proposed in our two-component model as a dominant force in the positional preference in *Drosophila*, (b) the underestimation of the occupancy of the outer zone of the first double spiral arena may indicate that distal cues and/or global knowledge of the arena may impact positional preference. Additional modeling and behavioral experiments are needed to resolve this issue.

### An Irregular arena

**Figure 8:**
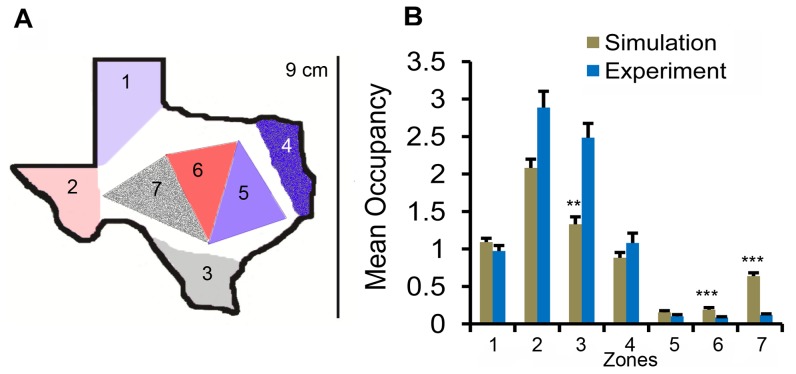
Positional preferences from experiments and simulations in Texas arena. We examined the preference of seven spatial zones inside the arena: four corners (zones 1, 2, 3, and 4) and three internal regions (zones 5, 6, and 7). Zone 5 is a triangle connecting three cities: Dallas, San Antonio and Houston. Zone 6 connects Dallas, San Antonio and Abeline, while zone 7 connects San Antonio, Abeline and Fort Stockon. The simulations captured several trends similarly to the experiments, but underemphasized the responses to the preferred areas and overemphasized the less preferred - especially zone 7. The asterix indicates significant difference between simulations and experiments (*: 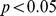, **: 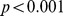, and ***: 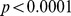).

To further examine the predictive power of the two-component model, we compared the movement of *Drosophila* inside an irregular arena in the shape of Texas to the trajectories from the model (Figure 8). The movement parameters used in the spiral arenas were also used to characterize the simulated trajectories in this irregular arena. In the Texas arena, there were no internal walls but the boundary had acute-angled corners. We examined the occupancies from simulations and experiments with Canton-S males in seven different zones (four corners: zones 1, 2, 3, and 4; three internal regions: zones 5, 6, and 7) as shown in Figure 8. The simulations captured similar trends of occupancy in these zones, but significantly underemphasized a preferred corner and overemphasized two of the less preferred internal zones (zone 1: 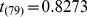, 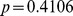; zone 2: 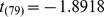, 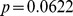, zone 3: 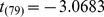, 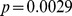, zone 4: 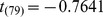, 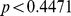, zone 5: 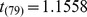, 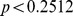, zone 6: 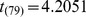, 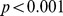, zone 7: 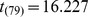, 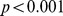). These data show that *Drosophila* spent more time in the acute-angled corners than predicted by the simulations. Interestingly, in the square arena wild type Canton-S spent more time in the corner than predicted by the two-component model, although this was not signficantly different (Figure 2). It is possible that the external corners in the square arena also have qualities not accounted for in the model, but these features do not have a strong enough effect to cause a statistical difference between the trajectories of wild type *Drosophila* and those obtained from the model (Figure 2).

### Examination of two-component model with mutant genotypes

We next examined the two-component model using four distinct fly lines with different defects in perception or sensory integration; these lines display different movement patterns within a circular open field arena. Flies with altered visual processing and wall-following behaviors are expected to exhibit modified 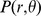 and positional preference in the different arenas. If movements of flies are completely described by the rules in the two-component model, we expect that simulations using altered parameters will give similar positional preferences to that of the fly with altered behaviors. Alternatively, the mutants may be used to uncover underlying complexity in the behavioral processes descibed by the rules, by specifically eliminating a process required to “obey" the rules. Four different mutants were used: 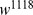, 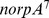, 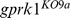 and 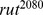 mutants. These mutants have altered wall-following behavior and magnitudes of exploration [16]. In the 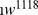 flies, the photoreceptor neurons are activated by tangential light, and as a consequence these flies have very poor visual contrast and cannot perform certain optimotor tasks [44]. Conversely, the 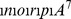 mutant flies are defective in phospholipase C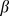, fail to perform a receptor potential, and are completely blind [45]. The 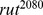 mutants are defective in a type I adenylyl cyclase and have pleiotropic learning defects [1], [46], [47]. Mutants in *rutabaga* have also been shown to display reduced wall-following behavior in open field arenas [41]. The 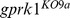 mutation is an amorphic allele of the *g protein receptor kinase 1* gene [48]. This gene is widely expressed in *Drosophila* and is known to regulate hedgehog signaling [48], [49]. Moreover, presumably due to its function in the termination of Rhodopsin signaling, decreases in *gprk1* function lead to increased visual responsiveness in *Drosophila* [50], which is likely to affect visual perception and acuity. We have found that both 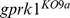 and 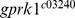 independent mutants display significant reductions in wall-following behavior in the open field arena (Figures S9, S10).

**Figure 9:**
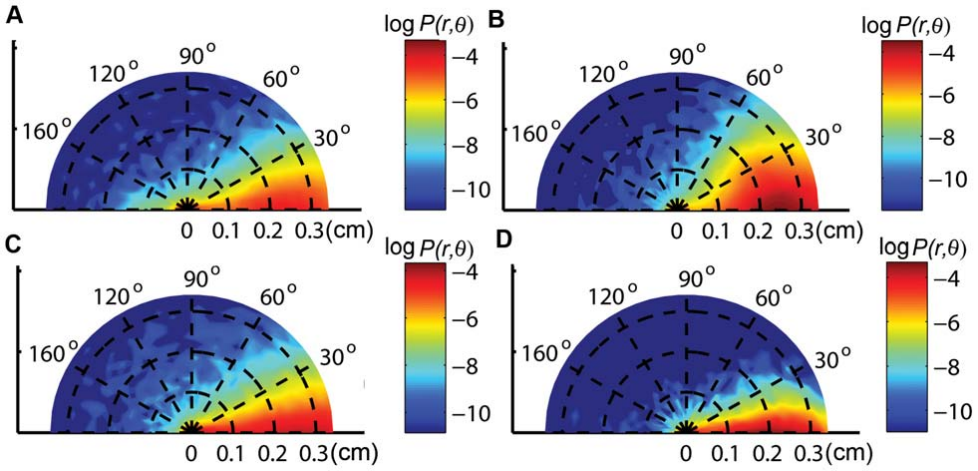
Directional persistence probability 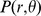
 **for four mutant flies.** 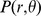 for 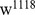, 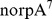, 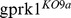 and 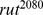 mutants are shown in panels **A**, **B**, **C** and **D**, respectively. The densities are shown in logarithmic scale. From the density plots, we found that 54.95%, 81.92%, 72.92%, 70.77% of the movements were restricted to an absolute turn angle of 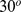 in 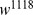, 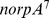, 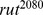, and 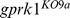, respectively. This shows that these mutants display different degrees of directional persistence in the central zone.

**Figure 10:**
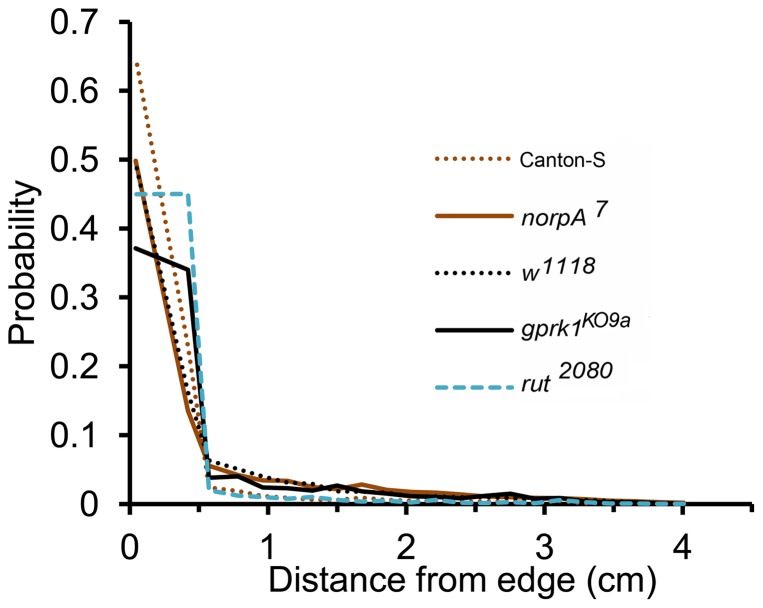
Radial distribution of four mutants and Canton-S flies in 4.2 cm circular arena. The mutants flies: 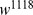, 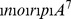, 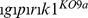 and 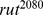 were found to spend 75%, 72%, 79% and 76% of their time within 0.6 cm of the arena boundary compared to 88% in Canton-S. There was a reduced wall preference in these mutants compared to Canton-S flies.

At least 100 individual flies for each of these 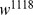, 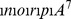, 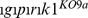 and 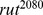 mutant genotypes were used in the 4.2 cm circular arena to estimate the directional persistence probability, 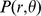 (Figure 9). The densities, 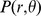, of each of these mutants were significantly different from wild type flies (Table S3). This indicates that these mutants displayed different trajectories from Canton-S flies in the central zone. The mutants also displayed different trajectories from each other in the central zone (Table S3). For each of the mutant flies, we computed the fraction of movements where the turn angle was less than 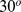, 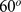 and 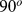 (Table S4). The 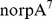 flies displayed the highest degree of directional persistence followed by 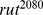, 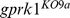 and 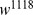. Interestingly, all of the mutants, except 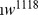, exhibited more directional persistence than the wild type flies (Table S4). The radial distributions of the four mutants (Figure 10) indicate that they have a reduced wall preference compared to wild type Canton-S flies. Nevertheless, the mutants still preferred the arena boundary over the open regions. The wall attraction for these four mutants were estimated using the same 0.6 cm annular region along the boundary to differentiate between edge and central zone and to define the comfort zone of the mutant flies (Figure S7). The 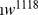 and 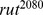 flies were found to have similar wall attraction (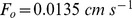), which was higher than that of 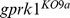 (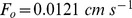), but lower than 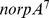 (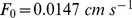). Hence, in these mutant genotypes the magnitude of directional persistence is uncoupled from the degree of preference for the arena's edge. The estimated parameters were used to simulate the movements of these mutants in two of the previously used arenas: internal corner and square arena.

**Figure 11:**
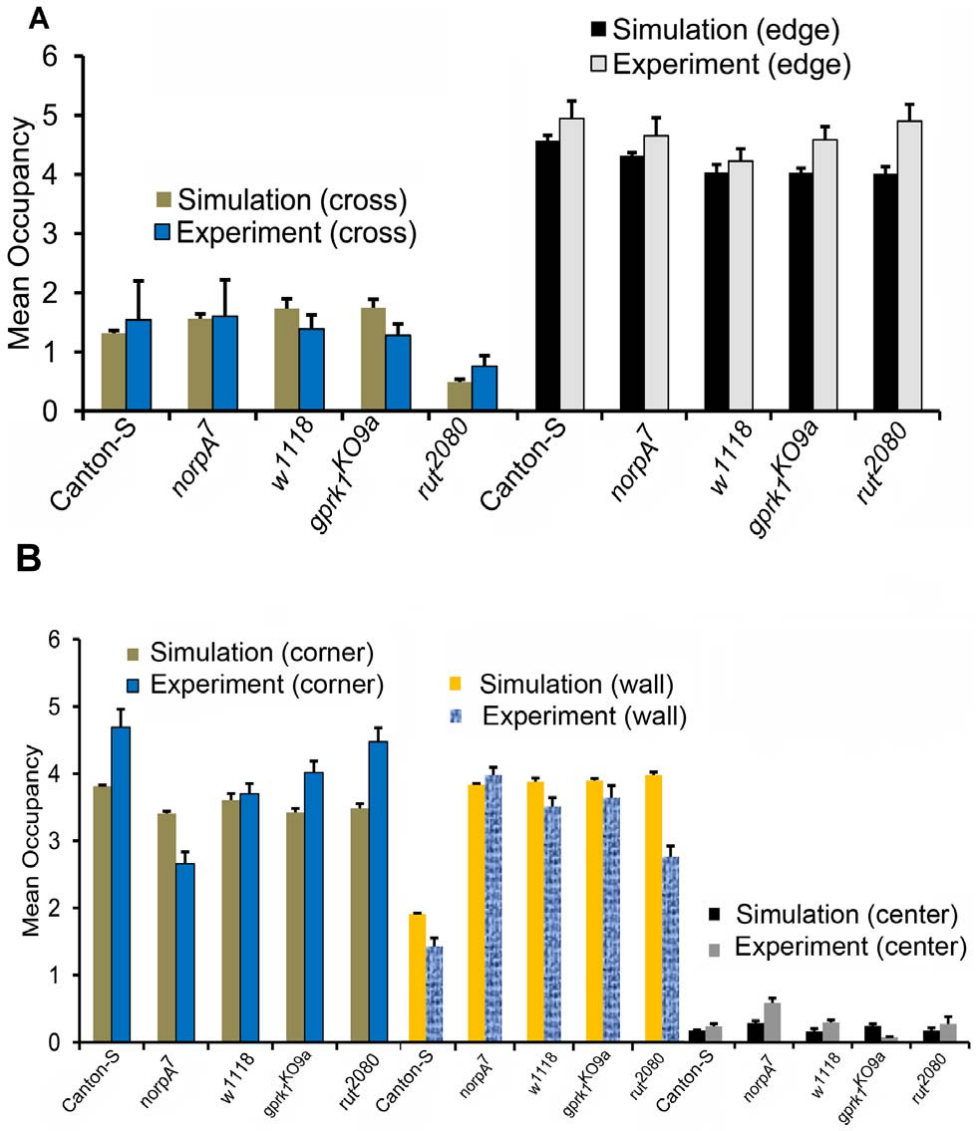
Positional preferences for four genotypes (experiments and simulations) inside a square and internal corner arenas. **A.** Mean occupancies for four mutant flies and wild type flies in different zones of the inner cross arena are shown. The 4 cm square sector at the center of the arena is the cross zone. The annular region of width 0.6 cm along the boundary comprised the edge zone. **B**. Mean occupancies for four mutant flies and wild type flies in different zones of 7.2 cm×7.2 cm square arena. The three spatial zones: corner, wall and center were divided as showin in Figure 2. The asterix indicates significant difference between simulations and experiments (*: 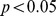, **: 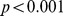, and ***: 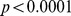). Significant differences between simulations and experiments are more prevalent in the square arena. Nevertheless, the simulations produced several trends in the occupancies which were similar to the experiments in both the arenas.

The spatial preferences of the four mutants in the internal corner arena were similar to those of our simulations (Figure 11A), except for occupancy of 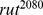 near the edge (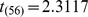, 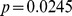). In the square arena, the occupancies from our simulations were statistically different from experiments in a number of cases (Figure 11B). Interestingly, the mean occupancy of the animals in the external corners increased as the visual acuity increased (lowest in 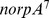 flies: 2.658±0.175 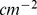; highest in Canton-S flies: 4.692±0.268 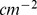). In the 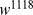 flies, which are poorly sighted, the two-component model was accurate for external corner preference (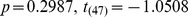). Both 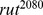 and wild type Canton-S flies, which have no visual defects, preferred external corners equally (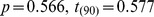). Most of the differences between the simulations and the mutants in the square arena can be accounted for by changes in the preference for external corners. This skewing towards corner preferences would also affect the occupancies of the wall and center zones in the square arena. This suggests that the external corners possess additional features which the two-component model might have missed. Also, intrinsic processes which contribute to corner preference may be disrupted. Since the two-component model fails mostly in predicting external corner preference in the mutants which have visual defects, there can be an important role of vision in forming external corner preference. This is lacking in the two-component model, even though we accounted for the difference in wall force in each mutant. Perhaps, different mathematical forms of wall attraction are required for flies with visual defects. Nevertheless, the model detected similar trends in the occupancy of mutant flies in both the internal corner and square arenas. These results indicate that wall attraction and directional persistence contribute to spatial preferences, but there may be other factors which contribute to positional preferences inside an arena.

### Model with a nonlinear force

There are three assumptions for the wall attraction in the two-component model. They are: (1) the magnitude of the attractive wall force decreases linearly with the distance between the fly and wall node, (2) the wall attraction is damping, and (3) the wall attractive force vanishes when the distance between the fly and a wall node is greater than 0.6 cm or radius of the comfort zone. It is possible that these assumptions imposed on the characteristics of the wall attraction contribute to the significant differences in occupancies between the two-component model and experiments in some arenas. To examine this, we considered two forms of nonlinear attractive force (equations 5 and 6) between the wall node 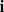 and fly.
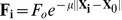
(5)

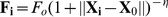
(6)


Equations 5 and 6 have nonlinear exponential and power law decay in the magnitude of the wall attraction with the increase in distance between the fly and wall node, respectively. The decay rates are determined by the parameters 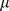 and 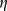, respectively. In these new force equations, the model fly experience attractive forces from wall nodes which lie beyond the comfort zone. We consider both damping and non-damping forces. For the damping force, the displacement 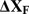 is 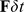. For the non-damping force, the displacement 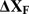 depends on the fly's displacement during the previous time step, 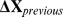, and is given by equation 7.
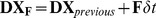
(7)


**Figure 12:**
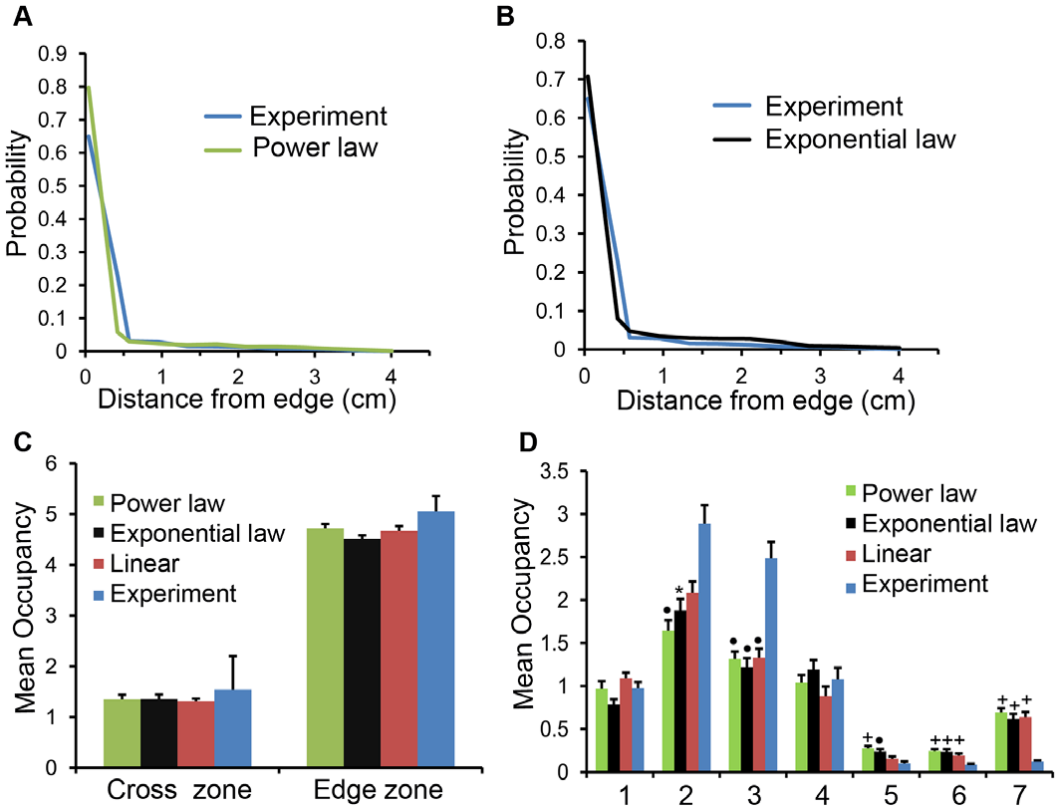
Positional preferences from experiments and two-component model with nonlinear wall forces in two arenas. **A**. Radial distribution of a wild type fly and simulations using a power law form of wall attraction, with 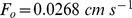 and 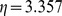, in the circular arena of radius 4.2 cm. Simulations using these values most closely matched the positional preference of wild type Canton-S. **B**. Radial distribution of a wild type fly and simulations using a exponential law form of wall attraction, with 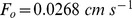 and 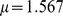, in the circular arena of radius 4.2 cm. Simulations using these values most closely matched the positional preference of wild type Canton-S. **C**. We examined the preference of two spatial zones (cross and edge zones as described in Figure 3) inside 4.2 cm circular arena with internal corners. There were no statistical differences in the occupancies from simulations (both exponential and power law decay wall attraction) and experiments for the two zones. **D**. We examined the occupancies in seven spatial zones in the Texas area as described in Figure 8. Both the two groups of simulations using exponential and power law forms of forces captured several trends similarly to the experiments, but underemphasized the responses to the preferred areas and overemphasized the less preferred - especially zone 7. The level of significant differences between simulations and experiments are indicated using *: 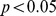, 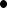: 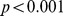, and +: 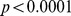.

For both the two forms of forces (Equations 5 and 6), we considered the same 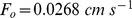 which was used for the two-component model with a damping linear local wall attraction. To determine the appropriate values for 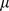 and 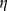, two groups of simulations were performed on the thermoplastic circular arena of radius 4.2 cm (Figure S11)- representing the power law and exponential nonlinear forces separately. In both the groups we used 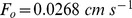 and the previously estimated directional persistence probability, 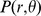. The simulations were performed separately for the damping and non-damping forces. In the case of nonlinear power law force, the radial distribution using non-damping force and 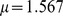 was closest to that from experiments (Figure 12A). In the case of nonlinear exponential law force, the radial distribution using non-damping force and 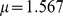 was closest to that from experiments 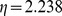 (Figure 12B). These two best cases for nonlinear wall attraction were used for subsequent analysis to test the accuracy of the two-component model obeying a nonlinear wall attraction.

To examine the accuracy of the two-component model with a nonlinear wall attraction, two groups of simulations were performed on two arenas (internal corner arena and Texas arena), one group using 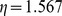 with nondamping force corresponding to the power law and the other using 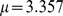 with damping force representing the exponential law. For both the groups, the occupancies in the different zones of the two arenas obtained from the simulations and experiments were compared (Figures 12C and 12D).

In the internal corner arena, there were no significant differences between the positional preferences from the simulations and the experiments (Figure 12C; power law - center: 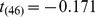, 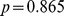, edge: 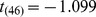, 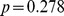; exponential law - center: 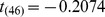, 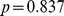, edge: 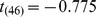, 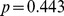). These data indicate that positional preferences of *Drosophila* inside the internal corner arena can be described by directional persistence and wall attraction which is nonlinear and nonlocal.

In the Texas arena, the simulations gave similar trends of occupancy as the experiments but there were some significant differences between the simulations and the experiments (Figure 12D). Both groups of simulations overpredicted the occupancies in the central zones especially zone 7 (Figure 12D; power law - zone 7: 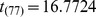, 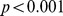 and exponential law - zone 7: 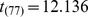, 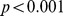), and underpredicted the occupancies in two boundary zones (Figure 12D; power law - zone 2: 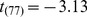, 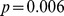; zone 3: 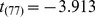, 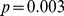 and exponential law - zone 2: 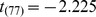, 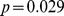; zone 3: 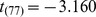, 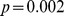). These data show that *Drosophila* spent more time in the acute-angled corners than predicted by the simulations. It is possible that the flies respond in a more complex way to the irregular boundaries in the Texas arena. Such response may not be captured by a nonlinear and nonlocal wall attraction.

The two-component model generated similar positional preferences as *Drosophila* in inner cross arena for both linear or a nonlinear wall attraction. This indicates that form of the force is not important as long as 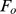 remains constant. The nonlinear forms of wall attraction did not improve the significant differences in the positional preferences between the model and experiments in the Texas arena. This indicates that three previous constraints on the nature of wall attraction (local, damping and linear wall attraction) are not responsible for the significant differences in positional preferences between the two-component model and *Drosophila*.

## Discussion

We propose that the positional preference of *Drosophila* within open field arenas is primarily driven by both a directional persistence in movements and a local wall attraction. We have used a phenomenological model to support this hypothesis. Our model limits movements through two simple mathematical rules that define the trajectories and local wall attraction. Trajectories following this two-component model exhibit similar positional preferences to *Drosophila* in a variety of arenas. Our two-component model captures several trends of spatial preferences even in arenas with complex geometries.

### *Drosophila* display strong directional persistence

The first local rule that drives movements in the two-component model is a directional persistence probability function that controls step size and turn angle. The presence of a strong directional persistence has also been previously reported during movements of cells, insects including *Drosophila*, birds and mammals [28], [38], [51], [52]. Similar to these previous reports, we find that both wild type and mutant flies have a strong directional persistence in regions away from the wall. This tendency for forward trajectories is a central component of *Drosophila* locomotion. Mathematical models of animal locomotion in open spaces have attempted to introduce directional persistence by using non-Gaussian turn angle distributions such as wrapped Cauchy [24], [40] and Von Mises distributions [42]. Such analytical models have shown that when animals have no information about their targets, the degrees of directional persistence in trajectories can alter the efficiency in finding targets [24], [40]. On the other hand, predator avoidance models show that straight trajectories have greater success against distant and slow moving predators, while rapid more convoluted trajectories have greater fitness against a close and fast predator [53]. Hence, it is possible that directional persistence plays an important role in an animal's fitness through increased chance of escape from predators and efficient foraging in a novel environment.

We also demontrate that step size and turn angle in *Drosophila* are interdependent. We find that flies tend to move slower while making large turn angles. There can be other factors that affect turning behavior which require the fly to slow down; examples may include sensory inputs from compound eyes, damping between body structures and air, and physical constraints [32]. For example, when walking, turns of less than 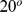 can be made by altering step length; however, while making large turn angles, a walking fly turns around its abdominal tip, and finally it increases its forward velocity [32]. Using legs and other body parts, *Drosophila* display a hierarchy of turning methods [32]. The variability in turn angles may arise from the motivational state of the fly, since it is not likely that changes are due to exhaustion or changes in external conditions such as temperature and illumination [32]. For example, large turn angles through rotation superimposed with translation followed by pure translation describe escape behavior in the cockroach [32].

### Wall attraction

The second local rule that drives movements in the two-component model is a local wall attraction. It had been previously shown that a prevalence of straight trajectories with a failure to make frequent sharp turns would promote a high occupancy at the boundaries of arenas [30]. But, persistent forward trajectories alone were insufficient to account for the behavior of *Drosophila* in an hourglass-shaped arena [29]. Specifically, in the absence of an attractive force towards walls, persistent forward motion could not account for wall-following behavior along convex walls which required the flies to make large turn angles. This means that the walls offer some attractive features to the flies. We have found that local wall attraction with directional persistence can produce spatial occupancies similar to *Drosophila* in a variety of arenas. The concordance between the behaviors of wild type Canton-S flies and that of the two-component model strongly indicate that a local wall attraction contribute to positional preferences in flies. In the two-component model, the force is contributed only from walls which lie in front of the fly and in close proximity to the fly. We have modeled nature of the wall attraction as a local interaction with walls to test the extent of use of proximal investigation by flies for positional preference. Even though we have described the wall attraction mathematically, this wall affinity should have intrinsic and external motivations. We also show that by keeping the value of 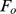 constant and imposing a nonlinear decay in the degree of wall attraction between the fly and wall as the distance between the two increases, we can also generate similar positional preferences as *Drosophila* in the internal corner arena. This indicates that as long as 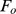 remains same, the form of the force is irrelevant for producing similar positional preferences as *Drosophila*.

 The presence of a local wall force can help explain the behavior of flies in several of our arenas, but the exact nature of this attraction remains unknown. Flies exhibit both walking along walls and on walls [29], [35]. Our model does not differentiate between these two behaviors; even after excluding instances of walking on walls, flies exhibit wall-following behavior along convex boundaries in an hourglass-shaped arena [29]. This strongly suggests that walking on walls alone is not sufficient to account for the attractive wall force in the two-component model. It is not clear if walking on walls and walking near walls are both driven by the same motivation, or if they represent complex and perhaps context-specific responses to the immediate environment. It has been suggested that avoiding the arena floor and walking on walls might make the flies less vulnerable to predation [35]. Another hypothesis is that flies which are walking on vertical surfaces are more likely to encounter odors from food sources than are flies that are walking on the floor of the arena [35]. Yet another suggested possibility is that flies that are walking on walls are more likely to jump and hence might use vertical walls as a launching platform [35]. The walls offer features which attract the flies to either walk on or along them. These hypotheses are not mutually exclusive, and the attraction demonstrated by *Drosophila* may be driven by a complex of behavioral processes.

### *Drosophila* use both proximal and distant cues

Since the two-component model replicates several different degrees of time-averaged positional preferences exhibited by the flies inside a variety of arenas (circle, square, concentric and internal corner) and captures several trends of spatial occupancies in spiral and Texas arenas, the two local rules we propose appear to be important factors contributing to positional preferences inside an open field arena. This strongly suggests that flies rely primarily on proximal cues while moving inside an arena. Our conjecture is consistent with previous work that found a fly's behavioral response to objects depends on the intrinsic properties of each object and not a relative assessment to other nearby objects [35]. If given a choice, flies preferentially approach the closest object; a judgment made using the motion parallax of the object's image on the retina and not expansion cues [33], [35], [54]. Therefore, proximal investigation contributes to flies' spatial preferences inside an arena.

The two-component model fails however, to completely and accurately capture all of the positional preferences displayed by *Drosophila* in diverse environments, suggesting that the model does not account for all of features of *Drosophila* behavior relevant for positional preference. In the two-component model, the interaction between the flies and the environment occurs only on proximal sites of the walls; the directional persistence probability density is independent of the geometry of the arena. In this model, animal locomotion within the arena is primarily determined by actions specified locally and with no significant consideration to global facets of an arena. The failure of the two-component model to capture the appropriate spatial occupancy in spiral and Texas arenas suggests that other features of *Drosophila* behavior, not included in the two-component model, exist which influence positional preference. We propose two features which the two-component model might have missed. The first possible feature may be a more complex, but still local, interaction with a wall edge than allowed in the two-component model. For example, the end of the walls located centrally in the spiral arenas may have an additional draw on the fly that could refract the trajectory. Similarly, it is possible that different portions of the boundary in the Texas arena offer different attractive forces depending on the tortuosity of the boundary. Even with a nonlinear wall attraction which was not local, the two-component model failed to generate similar positional preferences as *Drosophila* in the Texas arena. Different mathematical forms of wall force might have to be adopted at different portions of the arena to accurately model this response. The second possible feature is that distal cues and/or global knowledge of the arena may impact positional preference. This is supported by the similar positional preferences in the internal corner arena between *Drosophila* and two-component model where the wall attraction had contributions from distal sites. Insects learn their environment and probably form a spatial representation as they explore [55]–[58]. Recently, it has been demonstrated that *Drosophila* are capable of forming and retaining visual place memories [59]. Information of different portions of the arena gathered during locomotion may be used to create some global representation of the arena. Certain areas, such as the acute angled territories in the Texas arena, may evoke a safe haven for the fly, which can add to the attractiveness of the region. A fly seeking escape routes may move to the most distal zones in a spiral arena. It is possible that the positional preferences of *Drosophila* in different environments emerge from multi-faceted responses to both global representations and proximal features of the arena.

Finally, the two-component model fails to accurately predict corner preferences of certain mutant flies inside the square arena, while still capturing the trends in preferences. Interestingly, the model accurately predicts the preference for internal corners in these additional genotypes, suggesting that the external corners in the square arena possess additional features not accounted for in the two-component model. Since the two-component model can accurately account for the corner preference of wild type Canton-S, these mutant genotypes are likely disrupted in the processes that drive the external corner preference. Although these mutants all display significantly different directional persistence when away from the wall, the two-component model captures these differences. Hence, we propose that the failure of the two-component model results from differences between Canton-S and the mutant genotypes in the wall force component. Three of the mutant lines have visual defects [44], [45], [50]. The 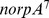 mutants are completely blind and spend the least time in the external corners of the square arena, while the normally sighted 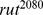 send more time in these corners than predicted by the two-component model. Although a direct role for vision in shaping wall force is an attractive hypothesis, this difference could also arise indirectly. Flies with reduced visual acuity display significantly more exploratory activity in open field arenas [16], [29], and this increased level of activation may inhibit them from settling into a corner to the same degree as normally sighted flies. Additionally, when flies are approaching an object they encounter the optical flow created by their motion with respect to the environment [60], [61]. Even in the presence of this optical flow, flies are able to move and orient themselves to prominent visual features [60], [61]. Neural circuits which are associated with vision are likely responsible for the capability of object fixation in *Drosophila*. In mutants with vision defects, this ability might be disrupted, and hence their response to external corners is different. Thus the failure to accurately model corner preference in visually impaired flies may expose a component of *Drosophila* behavior that affects positional preference, but is not explicitly covered in the two-component model.

In this paper we have presented the approach of combining phenomenological modeling and experimental data to demonstrate that local wall attraction and directional persistence are important components that contribute generally to positional preference in many different environments. Our work indicates that flies rely on proximal cues while moving inside an open field arena. Using more complex arenas, we demonstrate the presence of new environmental features that attract flies such as external corners and irregular features on the walls, or the possible use of a global mapping strategy for positional preference in an arena. Using four different mutant genotypes, we suggest that internal processes, such as visual processing, contribute to the formation of a positional preference.

## Supporting Information

Figure S1 **Position characteristics of a fly inside square arena.** Two experiments, each of 10 minute duration broken into 1 minute intervals, are in **A** and **B**. The spatial density P(x,y) of fly in **A** and **B** are shown in **C** and **D**, respectively in logarithmic scale. The density plots clearly indicate that the fly prefer the boundary.
(TIF)

Figure S2 **Position characteristics of a fly inside concenric circular arena.** Two experiments, each of 10 minute duration broken into 1 minute intervals, are in **A** and **B**. The spatial density P(x,y) of fly in **A** and **B** are shown in **C** and **D**, respectively in logarithmic scale. The density plots clearly indicate that the fly prefer the boundary.
(TIF)

Figure S3 **Position characteristics of a fly inside internal corner arena.** Two experiments, each of 10 minute duration broken into 1 minute intervals, are in **A** and **B**. The spatial density P(x,y) of fly in **A** and **B** are shown in **C** and **D**, respectively in logarithmic scale. The density plots clearly indicate that the fly prefer the boundary.
(TIF)

Figure S4 **Position characteristics of a wild type fly inside a hourglass arena.** Two experiments, each of 10 minute duration broken into 1 minute intervals, are represented in **A** and **B**. The spatial density, P(x,y), of flies in **A** and **B** are shown in **C** and **D**, respectively in logarithmic scale. The density plots clearly indicate that the flies prefer the boundary.
(TIF)

Figure S5
**Equal preference of the four quadrants by Canton-s flies in the circular arena of radius 4.2 cm.** The circular arena was divided into four zones of equal areas. The mean percentage of time spent in each of these zones are shown. There was no significant effect of the location of the quadrants on the percentage of time spent (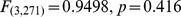). This shows that Canton-S flies had no biased preference for a quadrant.(TIF)

Figure S6
**Turn angle and speed are inter-dependent at different values of** 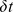 **.** We computed turn angle, 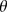, and step length, 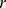, at two different values of 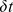 (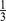 and 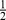 seconds). We used the computed turn angles and step lengths to obtain 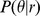 and 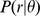 in the central zone of radius 4.2 cm arena. For 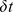 = 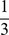 second, 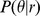 and 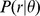 are shown in **A** and **B**, respectively. For 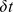 = 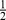 second, 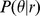 and 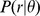 are shown in **C** and **D**, respectively. The plots clearly indicate that at different values of 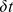, turn angle and speed are not independent of each other for *Drosophila*.(TIF)

Figure S7
**Wall force estimation for different genotypes from trajectories in a circular arena of radius 4.2 cm.** The radial distributions were computed for both the genotype and corresponding synthetic fly in a circular arena of radius 4.2 cm using histograms of bin size 0.2 cm. At each bin, the square of the difference between the two histograms was computed. The values from all the bins were added together to quantify the difference between experiment and simulation. This was done for different values of 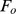 as shown. The value of 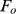 which gave the least difference was chosen to simulate the movement of the synthetic fly in other arenas. The values of 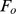 for different genotypes were: Canton-S = 0.0268 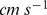, 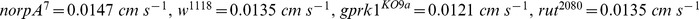.(TIF)

Figure S8
**Model parameters estimated from opaque thermoplastic circular arena of radius 4.2 cm.** There was no statistical difference between opaque and clear arenas for Radial distribution and directional persistent proability. The data from this circular arena was used to simulate the trajectories in the spiral and irregular arena. Here, 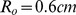 and 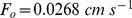.(TIF)

Figure S9
**Reduced arena edge preference by** 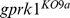 **flies in a circular arena of radius 4.2 cm.** The percentage of time spent in the edge zone by 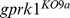 and control Canton-S flies are shown for each minute. There was a significant difference between the Canton-S and 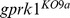 in the time spent in the edge zone (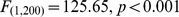).(TIF)

Figure S10
**Reduced arena edge preference by** 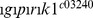 **flies in a circular arena of radius 4.2 cm.** The percentage of time spent in the edge zone by 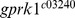 and control Canton-S flies are shown for each minute. There was a significant difference between the Canton-S and 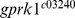 in the time spent in the edge zone (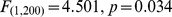).(TIF)

Figure S11
**Estimation of parameters in nonlinear wall forces.** Panels **A** and **B** show the difference between the radial distributions from experiments and two-component model using damping exponential and damping power law wall attraction, respectively. The decay parameters 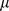 and 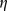 are varied in **A** and **B**, respectively. Panels **C** and **D** show the difference between the radial distributions from experiments and two-component model using non-damping exponential and non-damping power law wall attraction, respectively. The decay parameters 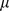 and 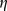 are varied in **A** and **B**, respectively. In all the four cases, 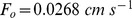 was used. The difference between the distributions from experiments and model was computed as described in Figure S5.(TIF)

Table S1

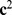
 **test to check if** 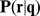 **is independent of** 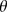 **.**
(PDF)

Table S2

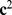
 **test to check if** 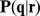 **is independent of r.**
(PDF)

Table S3 **Statistical comparison between the directional persistent probabilities of different genotypes.**
(PDF)

Table S4 **Percentage of movements in the central zone constrained by small turn angles.**
(PDF)
