# Correction: Modeling *Drosophila* Positional Preferences in Open Field Arenas with Directional Persistence and Wall Attraction

**DOI:** 10.1371/annotation/2ccf0a7e-4f7e-47e3-9aa2-b946fbf698b7

**Published:** 2012-10-15

**Authors:** Benjamin Soibam, Rachel L. Goldfeder, Claire Manson-Bishop, Rachel Gamblin, Scott D. Pletcher, Shishir Shah, Gemunu H. Gunaratne, Gregg W. Roman

There were errors in the placement of Figures 2-6.

The figure published as Figure 2 is actually Figure 6.

The figure published as Figure 3 is actually Figure 2.

The figure published as Figure 4 is actually Figure 3.

The figure published as Figure 5 is actually Figure 4.

The figure published as Figure 6 is actually Figure 5. 

