# Supplementary figures and images for: Modeling Drosophila Positional Preferences in Open Field Arenas with Directional Persistence and Wall Attraction
Source: PLoS One. 2012 Oct 10;7(10):e46570. doi: 10.1371/journal.pone.0046570 (PMC3468593; doi:10.1371/journal.pone.0046570)

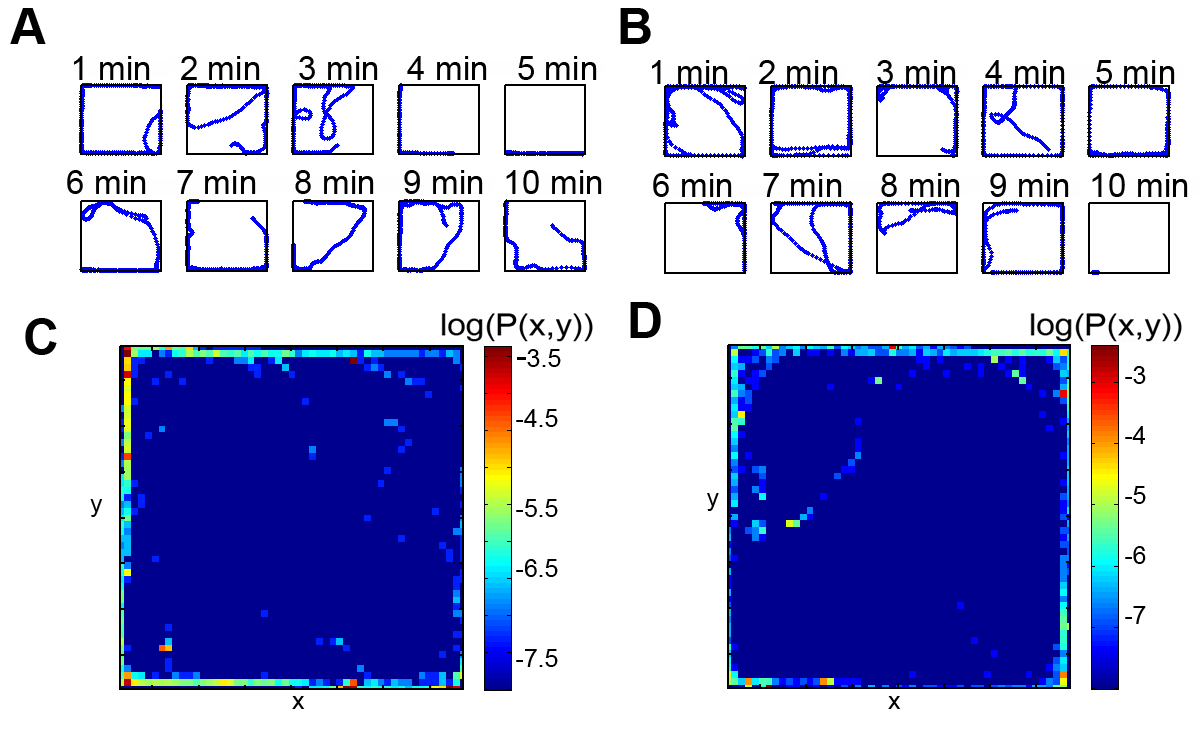

Supplement: Figure S1 — Position characteristics of a fly inside square arena. Two experiments, each of 10 minute duration broken into 1 minute intervals, are in A and B. The spatial density P(x,y) of fly in A and B are shown in C and D, respectively in logarithmic scale. The density plots clearly indicate that the fly prefer the boundary. (TIF) [file pone.0046570.s001.tif]

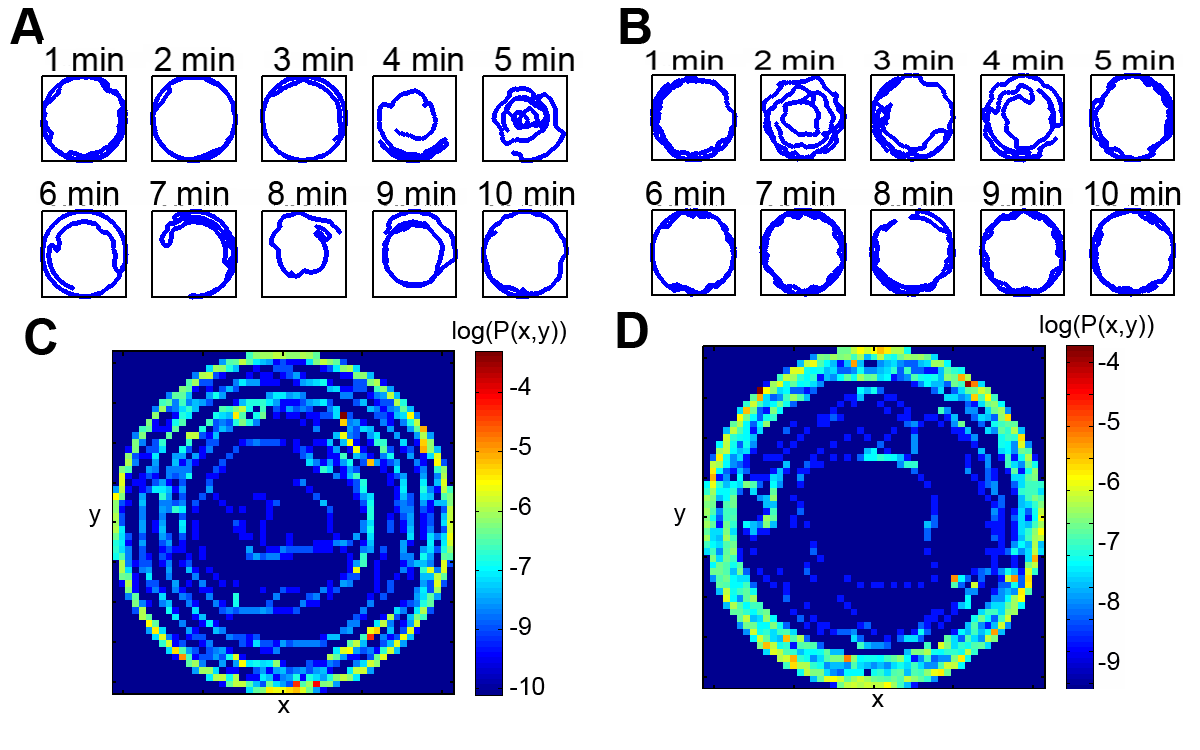

Supplement: Figure S2 — Position characteristics of a fly inside concenric circular arena. Two experiments, each of 10 minute duration broken into 1 minute intervals, are in A and B. The spatial density P(x,y) of fly in A and B are shown in C and D, respectively in logarithmic scale. The density plots clearly indicate that the fly prefer the boundary. (TIF) [file pone.0046570.s002.tif]

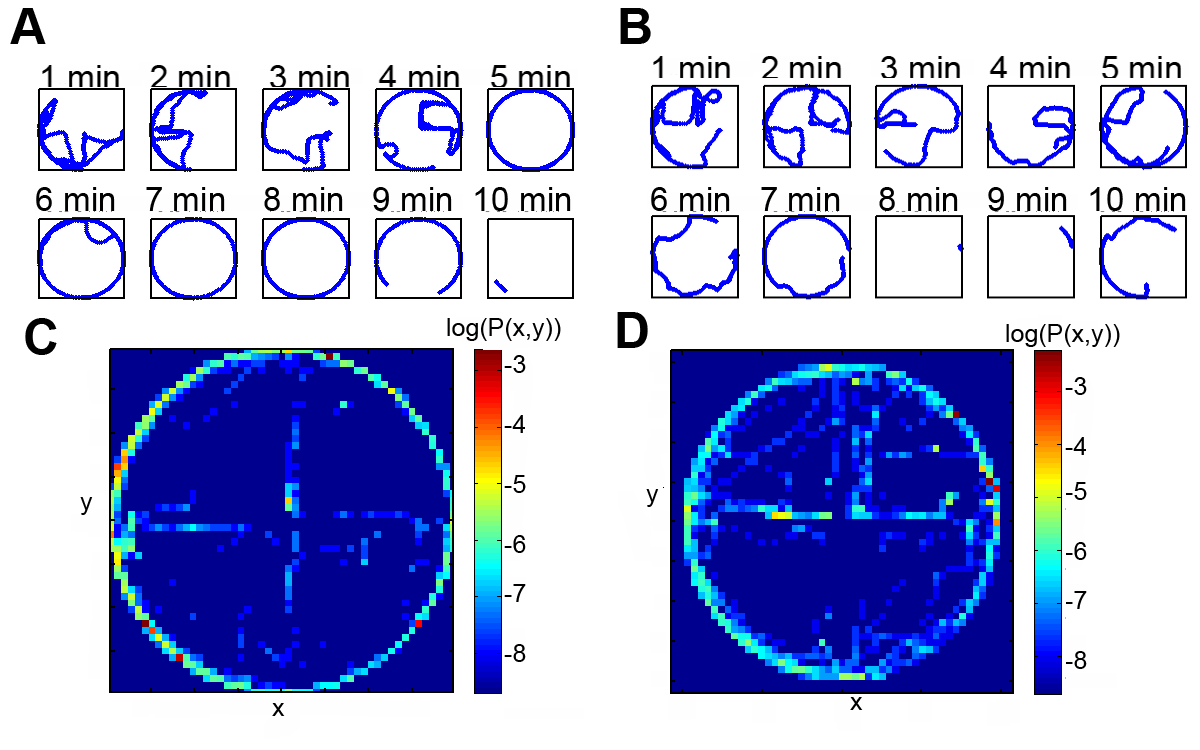

Supplement: Figure S3 — Position characteristics of a fly inside internal corner arena. Two experiments, each of 10 minute duration broken into 1 minute intervals, are in A and B. The spatial density P(x,y) of fly in A and B are shown in C and D, respectively in logarithmic scale. The density plots clearly indicate that the fly prefer the boundary. (TIF) [file pone.0046570.s003.tif]

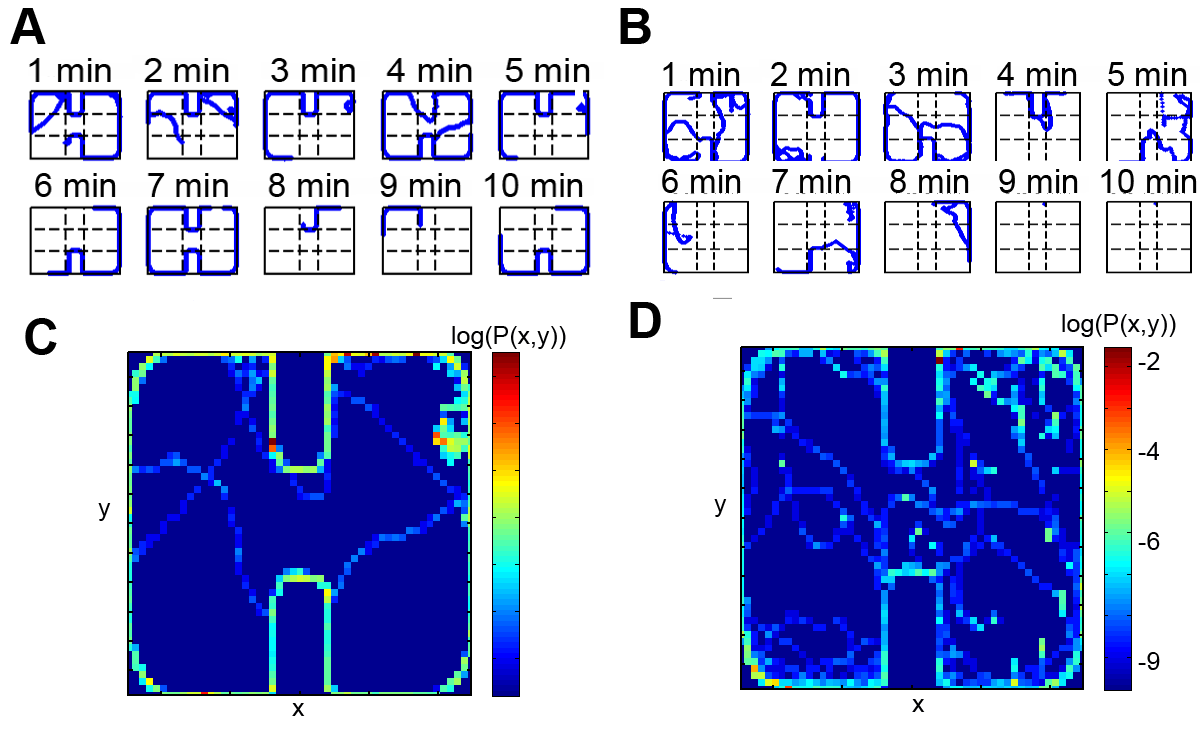

Supplement: Figure S4 — Position characteristics of a wild type fly inside a hourglass arena. Two experiments, each of 10 minute duration broken into 1 minute intervals, are represented in A and B. The spatial density, P(x,y), of flies in A and B are shown in C and D, respectively in logarithmic scale. The density plots clearly indicate that the flies prefer the boundary. (TIF) [file pone.0046570.s004.tif]

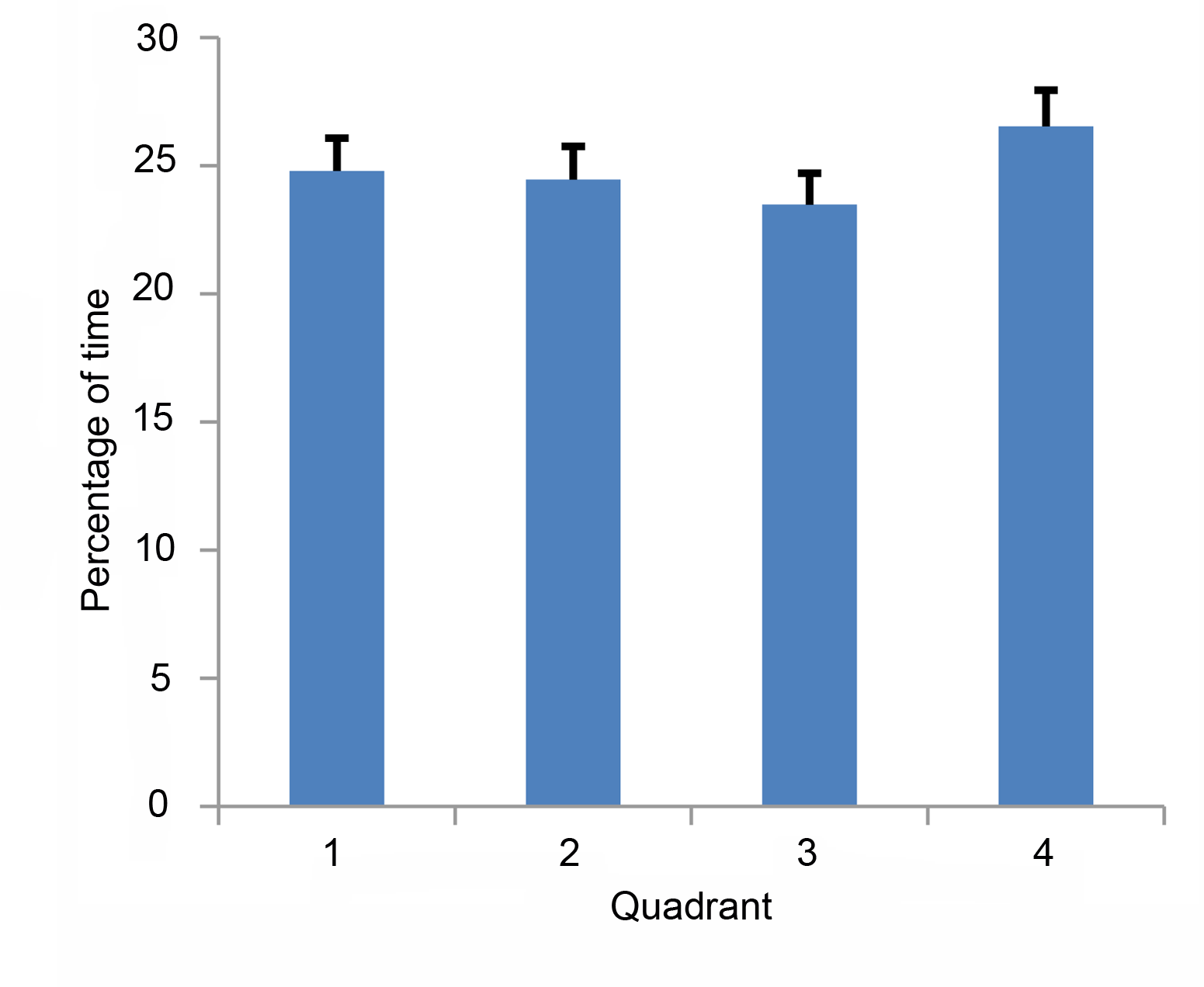

Supplement: Figure S5 — Equal preference of the four quadrants by Canton-s flies in the circular arena of radius 4.2 cm. The circular arena was divided into four zones of equal areas. The mean percentage of time spent in each of these zones are shown. There was no significant effect of the location of the quadrants on the percentage of time spent (). This shows that Canton-S flies had no biased preference for a quadrant. (TIF) [file pone.0046570.s005.tif]

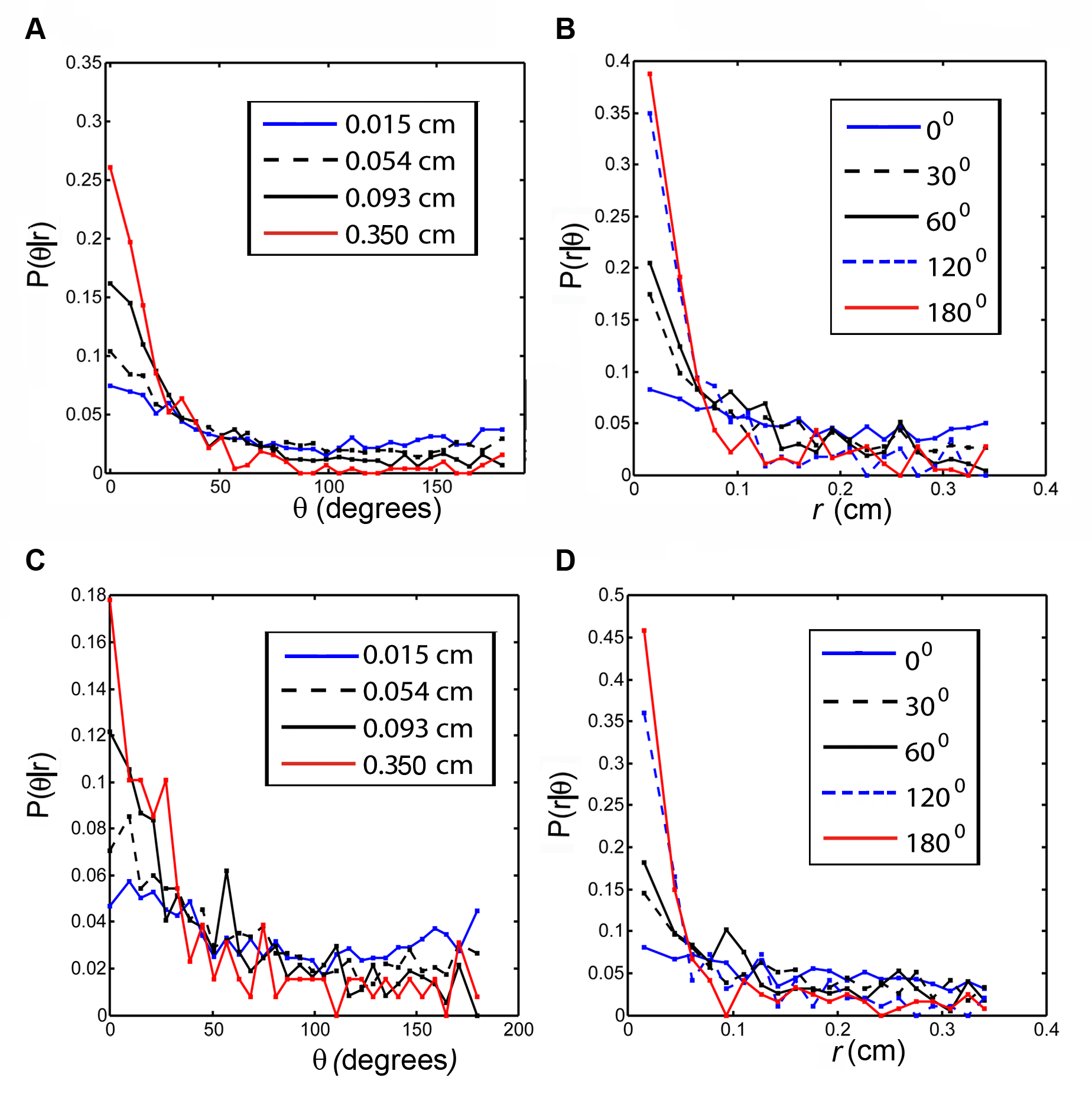

Supplement: Figure S6 — Turn angle and speed are inter-dependent at different values of . We computed turn angle, , and step length, , at two different values of ( and seconds). We used the computed turn angles and step lengths to obtain and in the central zone of radius 4.2 cm arena. For = second, and are shown in A and B, respectively. For = second, and are shown in C and D, respectively. The plots clearly indicate that at different values of , turn angle and speed are not independent of each other for Drosophila. (TIF) [file pone.0046570.s006.tif]

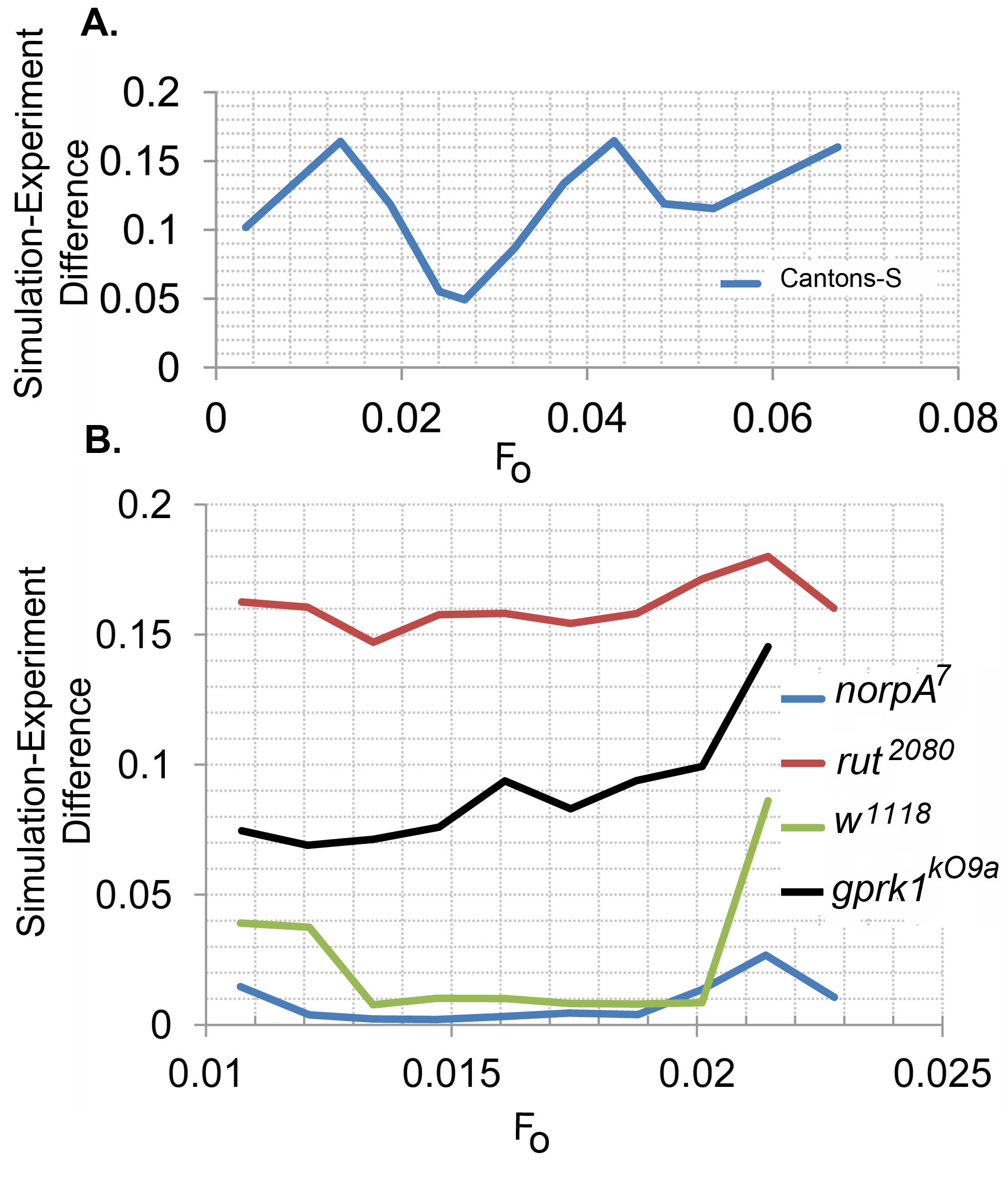

Supplement: Figure S7 — Wall force estimation for different genotypes from trajectories in a circular arena of radius 4.2 cm. The radial distributions were computed for both the genotype and corresponding synthetic fly in a circular arena of radius 4.2 cm using histograms of bin size 0.2 cm. At each bin, the square of the difference between the two histograms was computed. The values from all the bins were added together to quantify the difference between experiment and simulation. This was done for different values of as shown. The value of which gave the least difference was chosen to simulate the movement of the synthetic fly in other arenas. The values of for different genotypes were: Canton-S = 0.0268 , . (TIF) [file pone.0046570.s007.tif]

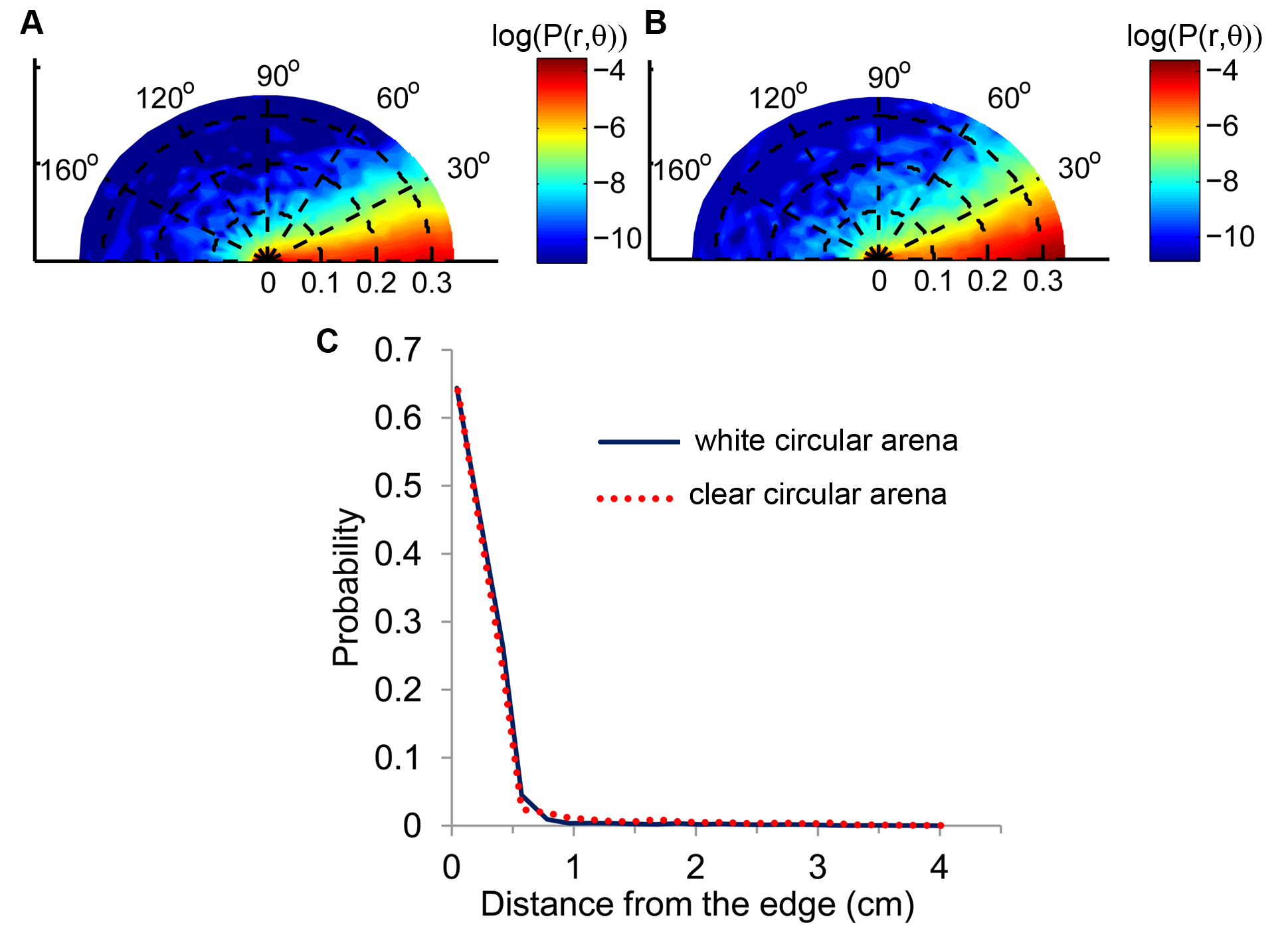

Supplement: Figure S8 — Model parameters estimated from opaque thermoplastic circular arena of radius 4.2 cm. There was no statistical difference between opaque and clear arenas for Radial distribution and directional persistent proability. The data from this circular arena was used to simulate the trajectories in the spiral and irregular arena. Here, and . (TIF) [file pone.0046570.s008.tif]

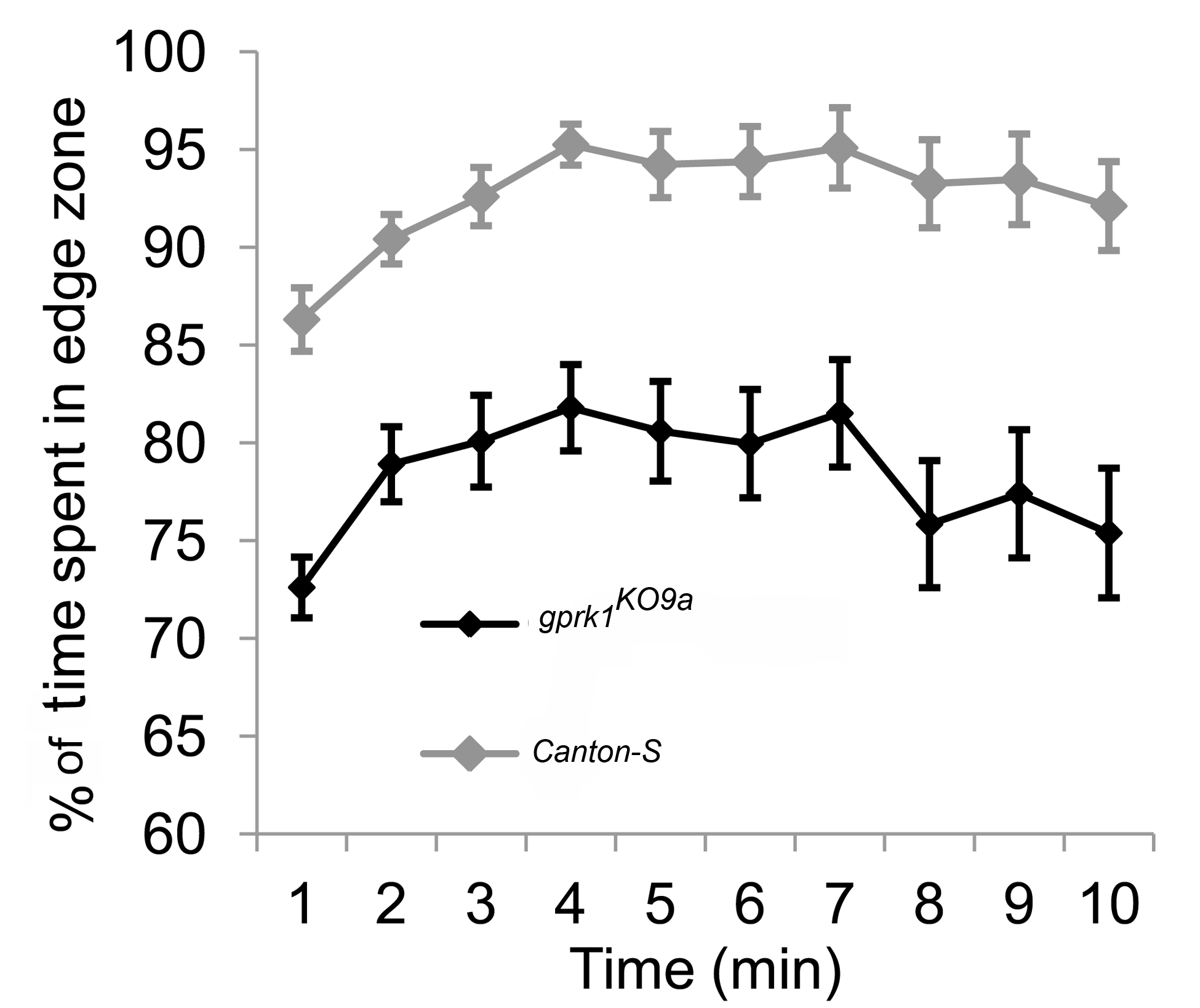

Supplement: Figure S9 — Reduced arena edge preference by flies in a circular arena of radius 4.2 cm. The percentage of time spent in the edge zone by and control Canton-S flies are shown for each minute. There was a significant difference between the Canton-S and in the time spent in the edge zone (). (TIF) [file pone.0046570.s009.tif]

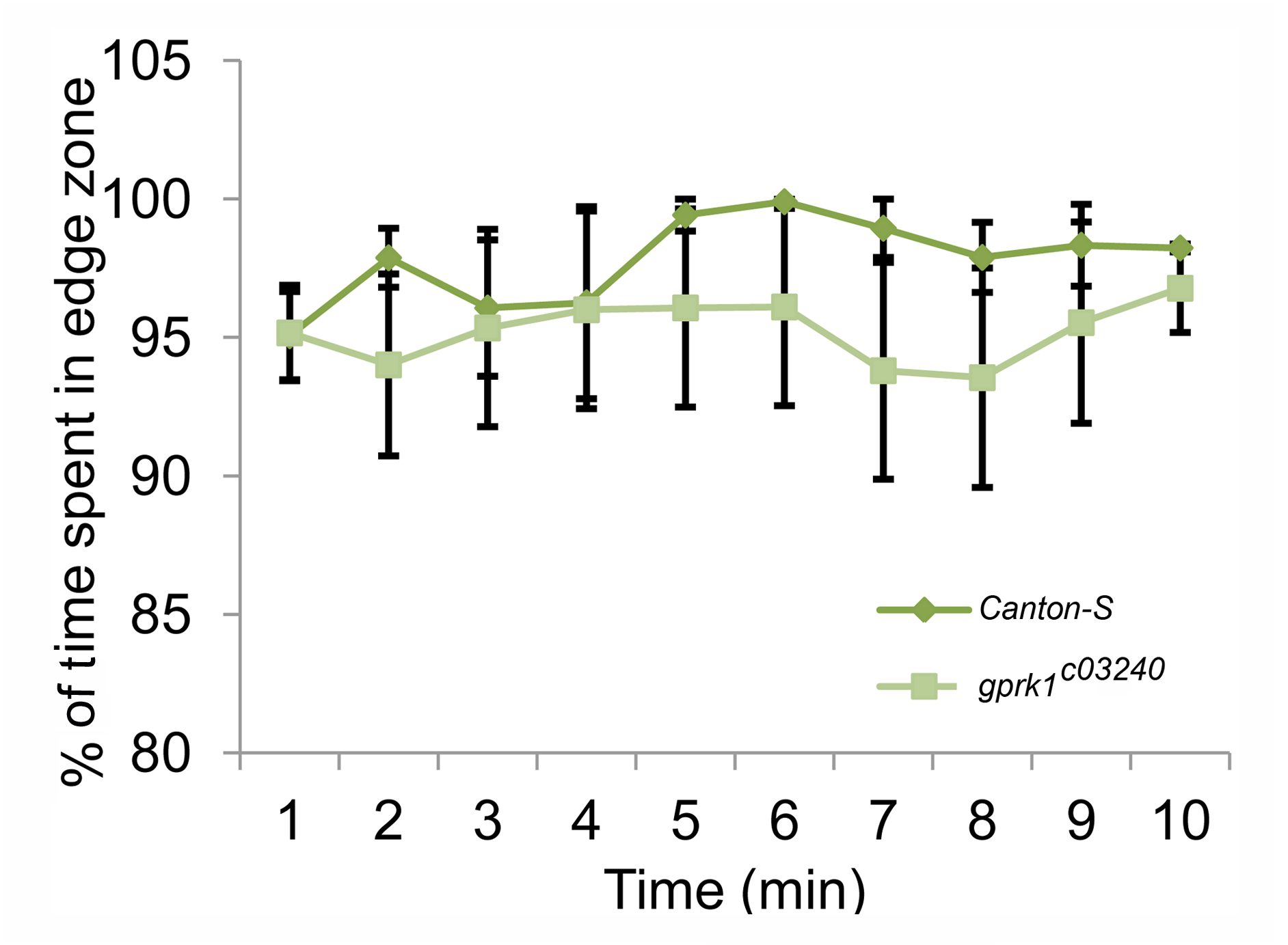

Supplement: Figure S10 — Reduced arena edge preference by flies in a circular arena of radius 4.2 cm. The percentage of time spent in the edge zone by and control Canton-S flies are shown for each minute. There was a significant difference between the Canton-S and in the time spent in the edge zone (). (TIF) [file pone.0046570.s010.tif]

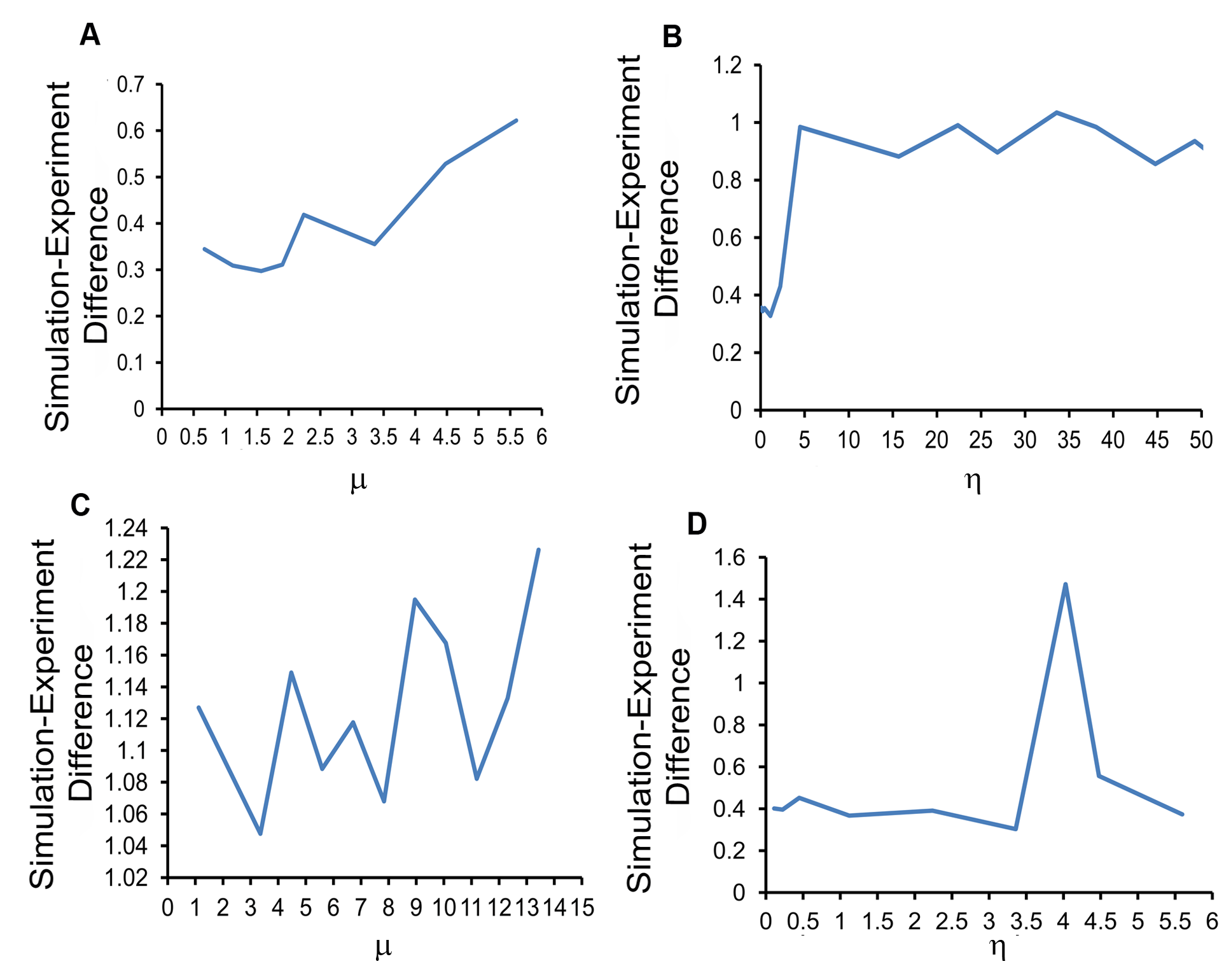

Supplement: Figure S11 — Estimation of parameters in nonlinear wall forces. Panels A and B show the difference between the radial distributions from experiments and two-component model using damping exponential and damping power law wall attraction, respectively. The decay parameters and are varied in A and B, respectively. Panels C and D show the difference between the radial distributions from experiments and two-component model using non-damping exponential and non-damping power law wall attraction, respectively. The decay parameters and are varied in A and B, respectively. In all the four cases, was used. The difference between the distributions from experiments and model was computed as described in Figure S5. (TIF) [file pone.0046570.s011.tif]
