# Supplementary material for: Modeling Drosophila Positional Preferences in Open Field Arenas with Directional Persistence and Wall Attraction
Source: PLoS One. 2012 Oct 10;7(10):e46570. doi: 10.1371/journal.pone.0046570 (PMC3468593; doi:10.1371/journal.pone.0046570)
Supplement: Table S1 — test to check if is independent of . (PDF) [file pone.0046570.s012.pdf]

Table S1: χ^2 test to check if $\mathbf{P(r|\theta)}$ is independent of θ. The distributions for five different θ were compared using a χ^2 test. The table indicates the χ^2 statistic values. For all comparisons, $df = 30$, $p < 0.0001$. Each column and row represents a turn angle as indicated.

	$\theta = \mathbf{0^o}$	$\theta = \mathbf{30^o}$	$\theta = \mathbf{60^o}$	$\theta = \mathbf{120^o}$	$\theta = \mathbf{180^o}$
$\theta = \mathbf{0^o}$		0.1481e+008	0.3877e+008	0.5216e+008	0.6137e+008
$\theta = \mathbf{30^o}$	0.2195e+008		0.0879e+008	0.1800e+008	0.2509e+008
$\theta = \mathbf{60^o}$	0.8170e+008	0.1250e+008		0.0275e+008	0.0664e+008
$\theta = \mathbf{120^o}$	1.3410e+008	0.3133e+008	0.0323e+008		0.0114e+008
$\theta = \mathbf{180^o}$	1.3958e+008	0.3970e+008	0.0753e+008	0.0105e+008	
