# Supplementary material for: Modeling Drosophila Positional Preferences in Open Field Arenas with Directional Persistence and Wall Attraction
Source: PLoS One. 2012 Oct 10;7(10):e46570. doi: 10.1371/journal.pone.0046570 (PMC3468593; doi:10.1371/journal.pone.0046570)
Supplement: Table S2 — test to check if is independent of r. (PDF) [file pone.0046570.s013.pdf]

Table S2: χ^2 test to check if $\mathbf{P}(\theta|\mathbf{r})$ is independent of r. The distributions for five different step size were compared using a χ^2 test. The table indicates the χ^2 statistic values. For all comparisons, $df = 20$, $p < 0.0001$. Each column and row represents a step size as indicated in bold.

	**r = 0.015cm**	**r = 0.054cm**	**r = 0.093cm**	**r = 0.350cm**
**r = 0.015cm**		0.5767e+008	0.6215e+008	0.6390e+008
**r = 0.054cm**	1.7876e+008		0.0078e+008	0.0143e+008
**r = 0.093cm**	1.7468e+008	0.0087e+008		0.0035e+008
**r = 0.350cm**	1.5588e+008	0.0159e+008	0.0032e+008	
