# Supplementary material for: Modeling Drosophila Positional Preferences in Open Field Arenas with Directional Persistence and Wall Attraction
Source: PLoS One. 2012 Oct 10;7(10):e46570. doi: 10.1371/journal.pone.0046570 (PMC3468593; doi:10.1371/journal.pone.0046570)
Supplement: Table S3 — Statistical comparison between the directional persistent probabilities of different genotypes. (PDF) [file pone.0046570.s014.pdf]

Table S3: Statistical comparison between the directional persistent probabilities of different genotypes. In the table, the first column indicates a pair of genotypes and the second column shows the result of the $\chi^{\mathbf{2}}$ test for comparing $P(r, \theta)$ for the two genotypes.

**Two genotypes**	**Statistical test**
gprk1^{KO9a}, w^{1118}	$\chi^2 = 16844$, $p < 0.001$, $df = 488$
gprk1^{KO9a}, $norpA^7$	$\chi^2 = 36520$, $p < 0.001$,df=490
gprk1^{KO9a}, rut^{2080}	$\chi^2 = 10347$, $p < 0.001$, $df = 382$
w^{1118}, $norpA^7$	$\chi^2 = 42468$, $p < 0.001$, $df = 474$
w^{1118}, rut^{2080}	$\chi^2 = 18147$, $p < 0.001$, $df = 372$
$norpA^7$, rut^{2080}<br>Canton-S, rut^{2080}	$\chi^2 = 86413$, $p < 0.001$, $df = 375$<br>$\chi^2 = 3.1289e + 007$, $p < 0.001$, $df = 573$
Canton-S, w^{1118}	$\chi^2 = 2.1182e + 009$, $p < 0.001$, $df = 555$
Canton-S, $norpA^7$	$\chi^2 = 1.7497e + 008$, $p < 0.001$, $df = 560$
Canton-S, $gprk1^{KO9a}$	$\chi^2 = 5.3981e + 007$, $p < 0.001$, $df = 539$
