# Supplementary material for: Modeling Drosophila Positional Preferences in Open Field Arenas with Directional Persistence and Wall Attraction
Source: PLoS One. 2012 Oct 10;7(10):e46570. doi: 10.1371/journal.pone.0046570 (PMC3468593; doi:10.1371/journal.pone.0046570)
Supplement: Table S4 — Percentage of movements in the central zone constrained by small turn angles. (PDF) [file pone.0046570.s015.pdf]

Table S4: Percentage of movements in the central zone constrained by small turn angles. The percentage was computed from the directional persistent probabaility. The first column indicate the genotype, the remaining columns indicate the % of movements less than turn angles 30°, 60° and 90°.

**Genotype**	**30°**	**60°**	**90°**
w^{1118}	57.84%	65.65%	70.32%
$norpA^7$	81.92%	89.42%	91.72%
rut^{2080}	72.92%	80.20%	84.33%
$gprk1^{KO9a}$	70.77%	79.53%	84.34%
